# Kinome screening identifies integrated stress response kinase EIF2AK1/HRI as a negative regulator of PINK1 mitophagy signaling

**DOI:** 10.1126/sciadv.adn2528

**Published:** 2025-05-09

**Authors:** Pawan K. Singh, Shalini Agarwal, Ilaria Volpi, Léa P. Wilhelm, Giada Becchi, Andrew Keenlyside, Thomas Macartney, Rachel Toth, Adrien Rousseau, Glenn R. Masson, Ian G. Ganley, Miratul M. K. Muqit

**Affiliations:** ^1^ MRC Protein Phosphorylation and Ubiquitylation Unit, School of Life Sciences, University of Dundee, Dundee DD1 5EH, UK.; ^2^ Division of Cellular Medicine, School of Medicine, University of Dundee, Dundee DD1 9SY, UK.

## Abstract

Loss-of-function mutations in the PINK1 kinase lead to early-onset Parkinson’s disease (PD). PINK1 is activated by mitochondrial damage to phosphorylate ubiquitin and Parkin, triggering mitophagy. PINK1 also indirectly phosphorylates Rab GTPases, such as Rab8A. Using an siRNA library targeting human Ser/Thr kinases in HeLa cells, we identified EIF2AK1 [heme-regulated inhibitor (HRI) kinase], a branch of the integrated stress response (ISR), as a negative regulator of PINK1. EIF2AK1 knockdown enhances mitochondrial depolarization–induced PINK1 stabilization and phosphorylation of ubiquitin and Rab8A. These results were confirmed in SK-OV-3, U2OS, and ARPE-19 cells. Knockdown of DELE1, an activator of EIF2AK1, produced similar effects. Notably, the ISR inhibitor ISRIB also enhanced PINK1 activation. In human cells with mito-QC mitophagy reporters, EIF2AK1 knockdown or ISRIB treatment increased PINK1-dependent mitophagy without affecting deferiprone-induced mitophagy. These findings suggest that the DELE1-EIF2AK1 ISR pathway is a negative regulator of PINK1-dependent mitophagy. Further evaluation in PD-relevant models is needed to assess the therapeutic potential of targeting this pathway.

## Teaser

EIF2AK1/HRI kinase negatively regulates PINK1 mitophagy.

## INTRODUCTION

Autosomal recessive mutations of genes encoding the mitochondrial protein kinase PTEN-induced kinase 1 (PINK1) and RING-IBR-RING (RBR) ubiquitin (Ub) E3 ligase Parkin are causal for familial early-onset Parkinson’s disease (PD) (*1*, *2*). Upon mitochondrial depolarization–dependent damage that can be induced in cells by chemical uncouplers [e.g., oligomycin/antimycin A (OA)], PINK1 undergoes stepwise activation with (i) protein stabilization, (ii) recruitment to the translocase of outer membrane (TOM) complex at the outer mitochondrial membrane (OMM), (iii) dimerization, (iv) trans-autophosphorylation of residue serine-228 (S228), and (v) stabilization of loop insertion 3 within its catalytic domain that enables the recognition of substrates Ub and Parkin (*3*–*8*). PINK1 directly phosphorylates an equivalent serine-65 (S65) residue found in Ub and Parkin, resulting in maximal activation of Parkin via a feed-forward mechanism triggering Ub-dependent removal of damaged mitochondria by autophagy (mitophagy) (*9*–*11*). We have also found that PINK1 activation leads to the phosphorylation of a subset of Rab guanosine triphosphatases including Rab8A at a highly conserved serine-111 (S111) residue located within its RabSF3 motif (*12*, *13*). The mechanism of Rab protein phosphorylation by PINK1 is indirect, suggesting the role of an unknown intermediate kinase (*12*, *13*). Furthermore, Rab8A S111 phosphorylation (Rab8A pS111) is a robust biomarker of PINK1 activation in cells and is abolished in human PINK1 knockout (KO) cells or cells expressing PD-associated mutants of PINK1 (*4*, *12*).

The integrated stress response (ISR) pathway consists of four branches: EIF2AK1/heme-regulated inhibitor (HRI), EIF2AK2/protein kinase double-stranded RNA-dependent (PKR), EIF2AK3/protein kinase R–like ER kinase (PERK), and EIF2AK4/general control non-derepressible-2 (GCN2), each responding to distinct stress signals (*14*–*17*). Upon sensing stress, ISR kinases phosphorylate eukaryotic translation initiation factor 2α (eIF2α), resulting in the down-regulation of protein synthesis and induction of the transcription factor ATF4 (activating transcription factor 4) (*14*). Recent pioneering work from the Jae and Kampmann labs has identified that, upon mitochondrial stress, the mitochondrial localized protein DAP3-binding cell death enhancer 1 (DELE1) is cleaved by the mitochondrial protease OMA1, and the C-terminal cleaved fragment of DELE1 is released into the cytosol, where it binds and activates EIF2AK1, leading to downstream ISR signaling (*18*, *19*). Previous studies have linked ISR with PD pathology (*20*) although no studies have directly assessed EIF2AK1 or DELE1 protein levels in PD tissues. Furthermore, no genetic variants in EIF2AK1 or DELE1 have been found as a risk factor for PD, although genetic studies have reported that EIF2AK3/PERK gene variants are a risk factor for the related neurodegenerative disorder, progressive supranuclear palsy (*21*).

An open question in the field is whether PINK1 is controlled by other kinase signaling pathways and networks. Herein, we have undertaken a genetic small interfering RNA (siRNA) screen targeting all known human Ser/Thr kinases using endogenous PINK1-phosphorylated Rab8A at S111 as a readout in HeLa cells and found that siRNA-mediated knockdown of the ISR kinase, EIF2AK1 (that encodes HRI), leads to a robust increase in Rab8A pS111 levels. We demonstrate that EIF2AK1 knockdown leads to increased stabilization and activation of PINK1 as measured by Ub Ser^65^ phosphorylation (Ub pS65) following mitochondrial depolarization. We further show that siRNA-mediated knockdown of DELE1 can also increase PINK1 stabilization and activation consistent with its role in activating EIF2AK1 under mitochondrial stress. These results are corroborated by the notable observation that the small-molecule ISR inhibitor ISRIB can also promote PINK1 stabilization and activation under conditions of mitochondrial depolarization similar to EIF2AK1 knockdown. Using the mito-QC mitophagy reporter (*22*, *23*), we also show that EIF2AK1 knockdown or ISRIB treatment enhances PINK1-dependent mitophagy but does not alter deferiprone (DFP)–induced mitophagy. Our results suggest that inhibition of the ISR by targeting DELE1-EIF2AK1 or by ISRIB and related analogs offer previously unidentified therapeutic strategies against PD.

## RESULTS

### Genetic siRNA screen for identification of kinases that regulate endogenous PINK1-dependent Rab8A phosphorylation

**Fig. 1.**
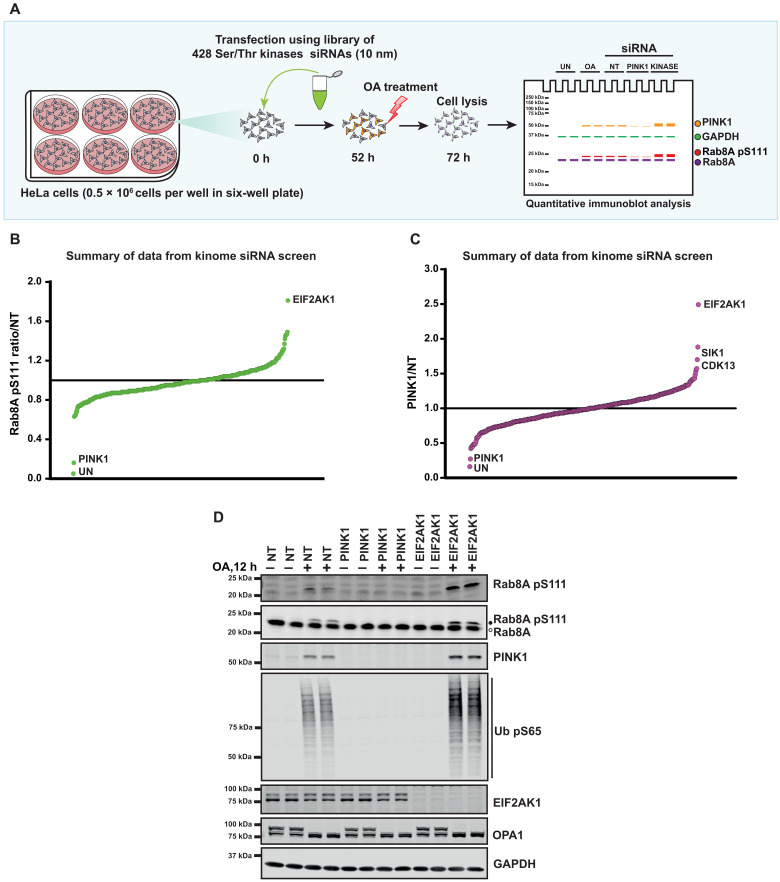
siRNA screen to identify kinases that regulate endogenous PINK1-dependent Rab8A phosphorylation. (**A**) Schematic of the siRNA knockdown screens used in this study. HeLa cells seeded in six-well plates at 0.5 × 10^6^ cells per well were transfected with siRNA pools (Dharmacon) for 72 hours targeting 428 Ser/Thr kinases with oligomycin (1 μM)/antimycin A (10 μM) (OA) treatment for the last 20 hours of siRNA targeting. Cells were lysed and immunoblotted for indicated antibodies and developed using the LI-COR Odyssey CLx Western blot imaging system for quantitative analysis. h, hours. (**B**) Summary of data from the kinome screen. The calculated ratio of Rab8A pS111/total Rab8A relative to non-targeting (NT) siRNA control, ranked from the highest increase in Rab8A phosphorylation to the strongest decrease (mean of the two replicates, SD values not shown on the chart, calculated using the LICOR Image Studio software, screening blots provided in figs. S1 to S4). (**C**) As in (B), the calculated ratio of PINK1/total GAPDH relative to NT siRNA control, ranked from the highest increase in PINK1 levels to the strongest decrease. (**D**) HeLa cells were transfected with siRNA for 72 hours with the top hit from the screen, EIF2AK1 along with the siRNA for PINK1 and NT siRNA control and OA treatment was done for the last 12 hours of siRNA targeting. Cells were then lysed and immunoblotted with Rab8A pS111, Rab8A, PINK1, Ub pS65, EIF2AK1, OPA1, and GAPDH antibodies and analyzed as described above.

To identify protein kinases that regulate endogenous PINK1-dependent Rab8A phosphorylation, we performed a siRNA screen targeting all Ser/Thr kinases in HeLa cells. We assembled and transfected a library of 428 siRNA pools (Horizon Dharmacon) targeting each kinase component for 72 hours with mitochondrial depolarization by adding 1 μM oligomycin/10 μM antimycin A (OA) in the last 20 hours of knockdown (to stabilize and activate PINK1) before cell lysis (Fig. 1A). To standardize the screen, we also included untransfected cells (UT), mock-transfected cells (no siRNA), and cells transfected with non-targeting (NT) siRNA as negative control and PINK1 siRNA as a positive control. As a readout of PINK1 kinase activity, we monitored S111 phosphorylation of Rab8A deploying a previously developed phospho-specific antibody that detects endogenous Rab8A pS111 by immunoblot analysis (*11*). Rab8A pS111 and total Rab8A levels were quantified in parallel using a multiplex immunoblot assay, and the ratio of Rab8A pS111 versus total Rab8A was used to calculate the degree of Rab8A phosphorylation in each cell lysate (Fig. 1A). Levels of total PINK1, glyceraldehyde-3-phosphate dehydrogenase (GAPDH), and optic atrophy 1 (OPA1) (cleaved OPA1 is a readout of mitochondrial depolarization) were also analyzed. In addition, immunoblotting of select kinase components of the siRNA library was performed to assess overall efficiency including BCR, TRIM28, RPS6KB2, BRD2, MAP3K7, CDC7, ROCK1, EEF2K, CAMK1D, GRK2, CSNK1A1, CSNK2A1, MAPK3, EIF2AK4, PBK/SPK, MAP3K5, MAP2K2, MAP2K6, and MAP2K7 (figs. S1 to S4). Notably, knockdown of EIF2AK1 enhanced Rab8A pS111 more than 1.8-fold (Fig. 1B). This was associated with a more than twofold increase in total PINK1 levels (Fig. 1C). While knockdown of SIK1 and CDK13 appeared to enhance PINK1 levels in the initial screen (Fig. 1C), these hits were not validated in follow-up knockdown analysis (fig. S5, A and B). Furthermore, we did not detect any hits that significantly reduced phospho-S111 Rab8A similar to PINK1 (Fig. 1B). Knockdown of several kinases mildly reduced Rab8A pS111 but were not significant (table S1).

To confirm that siRNA-mediated knockdown of EIF2AK1 enhances PINK1-mediated Rab8A pS111 levels, we generated a sheep polyclonal antibody against total human EIF2AK1 using recombinant full-length glutathione *S*-transferase (GST)–EIF2AK1 protein expressed and purified from *Escherichia coli* (fig. S5C). This antibody specifically recognizes EIF2AK1, but not the related EIF2AK4 by immunoblotting (fig. S5, D and E). Using this antibody, we were able to assess the degree of EIF2AK1 knockdown associated with PINK1 stabilization/activation under our siRNA conditions alongside NT and PINK1 siRNA (Fig. 1D). Notably, we did not observe any effect of EIF2AK1 siRNA on PINK1 levels in basal/unstimulated cells (Fig. 1D). Consistent with the screen results, upon mitochondrial depolarization induced by OA, we observed in EIF2AK1 knockdown cells a robust increase in PINK1 protein stabilization and Rab8A S111 phosphorylation (Fig. 1D). In addition, we observed that running lysates on 12% tris-glycine gels enabled visualization of an electrophoretic mobility band shift of Rab8A that accompanied Rab8A pS111, and this was abolished by PINK1 siRNA knockdown and enhanced by EIF2AK1 knockdown, providing a facile readout to monitor PINK1-dependent Rab8A pS111 (Fig. 1D). To confirm that the increased PINK1 stabilization is associated with increased PINK1 activation, we also measured levels of PINK1-dependent Ub pS65 and similarly found that EIF2AK1 siRNA knockdown increased levels of Ub pS65 (Fig. 1D). To verify the specificity of siRNA toward EIF2AK1, we performed similar experiments with independent pooled siRNA (Sigma-Aldrich) as well as single siRNAs (Dharmacon) for EIF2AK1 and observed that all led to enhanced PINK1 stabilization, and PINK1-phosphorylated Rab8A as determined by Rab8A band shift (fig. S6).

### Genetic validation studies confirm that specific knockdown of the ISR kinase, EIF2AK1, enhances PINK1 stabilization and activation in response to mitochondrial damage

**Fig. 2.**
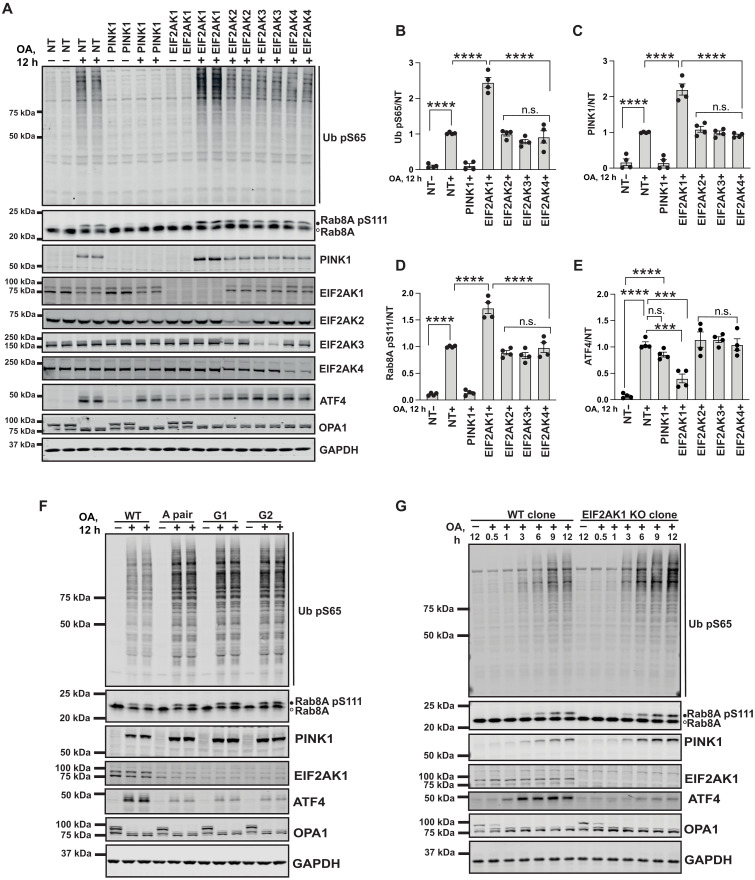
siRNA knockdown and CRISPR KO of the full-length EIF2AK1 in HeLa cells enhances PINK1 stabilization and activation. (**A**) HeLa cells treated with either non-targeting control siRNA pool (NT) or siRNA pool targeting PINK1, EIF2AK1, EIF2AK2, EIF2AK3, and EIF2AK4 for 72 hours were treated with OA in the last 12 hours. Cells were lysed and immunoblotted for Ub pS65, Rab8A, PINK1, EIF2AK1, EIF2AK2, EIF2AK3, EIF2AK4, OPA1, ATF4, and GAPDH, and the immunoblots were developed using the LI-COR Odyssey CLx Western blot imaging system. (**B**) Quantification of Ub pS65/GAPDH ratio normalized to the ratio in NT (+OA) samples using the Image Studio software. (**C**) Quantification of PINK1/GAPDH ratio normalized to the ratio in NT (+OA) samples. (**D**) Quantification of Rab8A pS111/Rab8A ratio normalized to the ratio in NT (+OA) samples. (**E**) Quantification of ATF4/GAPDH ratio normalized to the ratio in NT (+OA) samples. (**F**) Wild-type (WT) HeLa cells and HeLa cells transfected with three different sets of CRISPR-Cas9 EIF2AK1 guide RNAs (A pair, G1, and G2) were treated with OA for 12 hours. Cells were lysed and immunoblotted for Ub pS65, PINK1, Rab8A, EIF2AK1, ATF4, OPA1, and GAPDH and developed using the LI-COR Odyssey CLx Western blot imaging system [quantification shown as fig. S8 (A to C)]. (**G**) Selected EIF2AK1 KO clone A3 and WT HeLa cells treated with OA for 0.5, 1, 3, 6, 9, and 12 hours as above were analyzed for Ub pS65, PINK1, Rab8A, EIF2AK1, ATF4, OPA1, and GAPDH. Data information: [(B) to (E)] All data are means ± SEM; statistical significance is displayed as ***P ≤ 0.001; ****P ≤ 0.0001; n.s., not significant. *n* = 4 technical replicates (two biological replicates), one-way ANOVA, Tukey’s multiple comparisons test. h, hours.

We next compared the effect of EIF2AK1 siRNA knockdown with the knockdown of the other three eIF2α kinases: EIF2AK2, EIF2AK3, and EIF2AK4. Our observations revealed that only EIF2AK1 knockdown selectively enhanced PINK1 stabilization and activation as judged by increased levels of Ub pS65 and Rab8A pS111 (band shift) (Fig. 2, A and D). Furthermore, we also observed a robust induction of ATF4 with OA treatment that was abolished by EIF2AK1 knockdown but not following EIF2AK2/3/4 knockdown consistent with previous studies (Fig. 2A) (*18*, *19*). To determine whether regulation of PINK1 stabilization by EIF2AK1 is generalizable to other cells, we screened a panel of human cell lines including SK-OV-3 ovarian cancer cells that express high levels of endogenous PINK1 and Parkin (*4*, *24*), U2OS osteosarcoma cells that express moderate PINK1 and low levels of Parkin, and ARPE-19 retinal pigment epithelial cells that have moderate PINK1 expression and no Parkin similar to HeLa cells (fig. S7, A to C). Upon treatment of OA, we observed in all three cell lines that siRNA-mediated knockdown of EIF2AK1 enhanced PINK1 stabilization and activation as measured by Ub pS65 and Rab8A pS111, and this was also associated with inhibition of OA-induced ATF4 expression (fig. S7, A to C).

EIF2AK1/HRI was initially implicated in a specialized role in erythrocyte (red blood cell) development whereby it is activated upon falling heme concentrations via its N-terminal heme-binding domain (*25*, *26*). However, it is now established that EIF2AK1 is widely expressed and responds to multiple types of signals including mitochondrial dysfunction, oxidative stress, and heat shock (*15*, *18*, *19*). Mitochondrial dysfunction due to protein misfolding or mitochondrial depolarization induces up-regulation of the master transcriptional factor, ATF4, and expression of ATF4-target genes (*27*–*29*). Consistent with this, we observed a robust induction of ATF4 in OA-treated cells, which was abolished by EIF2AK1 knockdown (Fig. 2, A and E, and fig. S7, A to C).

To further validate the role of EIF2AK1, we used CRISPR-Cas9 gene-editing to knock out EIF2AK1 in HeLa cells. CRISPR guides (single guide1, guide 2, and guide pair A) were transfected into HeLa cells and immunoblot analysis of the CRISPR EIF2AK1 knockdown cellular pool lysates for all the guides demonstrated increased PINK1 stabilization and activation as measured by Ub pS65 and Rab8A pS111 (Fig. 2F and fig. S8, A to C). Following single-cell sorting and screening, we isolated two independent clones from guide pair A that were confirmed by sequencing and immunoblotting to be homozygous for loss-of-function mutations in the EIF2AK1 gene (fig. S9, A to E). EIF2AK1 KO clones, A2 and A3, were next treated with dimethyl sulfoxide or OA for 12 hours to induce mitochondrial depolarization. Immunoblot analysis of whole-cell lysates under basal conditions did not demonstrate any change in PINK1 levels or activity in EIF2AK1 KO cells compared to that in controls consistent with the siRNA studies (fig. S9F). However, upon OA treatment, there was a robust increase in PINK1 stabilization and activation as judged by Ub pS65 in both EIF2AK1 KO clones compared to that in the wild-type (WT) control cell (fig. S9F). We next determined the time course of PINK1 stabilization, Rab8A pS111 (band shift) and Ub pS65 in the EIF2AK1 clone A3 KO cells following OA treatment. We observed PINK1 protein levels becoming stable after 3 hours of OA treatment, in the WT control cells, associated with the accumulation of Ub pS65 and Rab8A pS111, and all three readouts were enhanced in EIF2AK1 KO cells, thereby validating the siRNA studies (Fig. 2G). We also observed a clear time-dependent up-regulation of ATF4 following OA treatment from 1 to 12 hours in WT cells (Fig. 2G). This up-regulation was abolished in EIF2AK1 KO cells, indicating that EIF2AK1 is the primary ISR kinase activated by mitochondrial dysfunction in our cell system (Fig. 2G).

We also noted that total EIF2AK1 levels were variably reduced in OA stimulated cells as compared to those in basal/untreated cells (e.g., Fig. 2A). To address whether this effect is mediated by the activation of PINK1, we compared EIF2AK1 protein levels in WT and PINK1 KO S-HeLa cells across a time course of OA stimulation. We observed a mild decrease in EIF2AK1 protein levels following OA treatment, and there was no difference between the WT and PINK1 KO cells (fig. S10). Therefore, OA-induced lowering of EIF2AK1 is PINK1 independent, and, recently, it was reported that silencing factor of the ISR (SIFI), an E3 ligase complex, mutated in early onset ataxia and dementia can regulate the degradation of EIF2AK1 to promote survival of cells undergoing mitochondrial import stress (*30*).

### DELE1-EIF2AK1 signaling relay negatively regulates PINK1

**Fig. 3.**
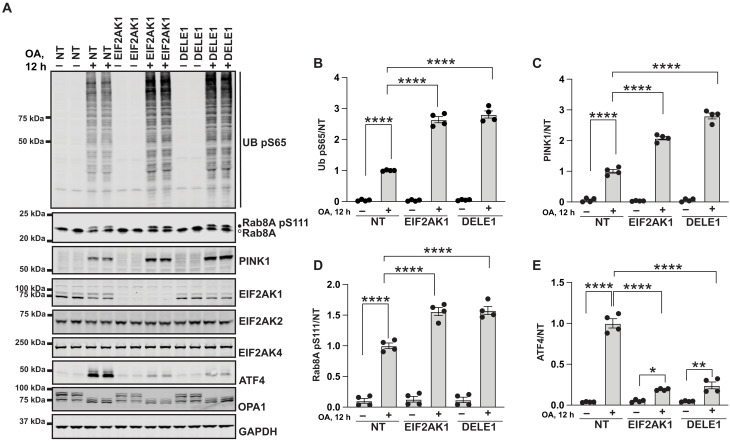
DELE1-EIF2AK1 signaling relay negatively regulates PINK1 stabilization and activation. (**A**) HeLa cells either treated with non-targeting control siRNA pool (NT), siRNA pool targeting EIF2AK1, or DELE1 for 72 hours were treated with OA in the last 12 hours. Immunoblot of Ub pS65, Rab8A, PINK1, EIF2AK1, EIF2AK2, EIF2AK4, ATF4, OPA1, and GAPDH. h, hours. (**B** to **E**) Quantification for Ub pS65/GAPDH, PINK1/GAPDH, Rab8A pS111/Rab8A and ATF4/GAPDH ratio for (A), normalized to the ratio in NT (+OA) samples. Data information: [(B) to (E)] All data are means ± SEM; statistical significance is displayed as *P ≤ 0.05; **P ≤ 0.01; ****P ≤ 0.0001; n.s., not significant. *n* = 4 technical replicates (two biological replicates), one-way ANOVA, Tukey’s multiple comparisons test.

Previous studies had revealed that, upon mitochondrial damage–induced activation of the mitochondrial protease OMA1, an inner mitochondrial membrane protein DELE1 is cleaved by OMA1, leading to accumulation of a shortened C-terminal fragment of DELE1 in the cytosol (*18*, *19*). This cleaved DELE1 fragment then oligomerizes in the cytoplasm and directly binds to and activates EIF2AK1, thereby initiating the ISR pathway (*18*, *19*). When exposed to oligomycin-induced mitochondrial stress, the cleaved DELE1 fragments are preferentially tagged by the SIFI complex and degraded in a manner similar to EIF2AK1 (*30*). To investigate the role of DELE1 in PINK1 activation and signaling, we performed siRNA-mediated knockdown of DELE1 alongside EIF2AK1 in HeLa cells (Fig. 3). We observed that following OA treatment, DELE1 knockdown led to a similar enhancement of PINK1 stabilization and activation as EIF2AK1 knockdown (Fig. 3, A to D). We also observed a substantial reduction in ATF4 levels in DELE1 knockdown cells, akin to the reduction seen in EIF2AK1 knockdown cells (Fig. 3, A and E). These findings highlight a major role of the DELE1-EIF2AK1-ISR signaling relay in negatively regulating mitochondrial damage–induced activation of PINK1.

### Transcriptional and translational up-regulation of PINK1 in EIF2AK1 knockdown cells during mitochondrial damage–induced stress

**Fig. 4.**
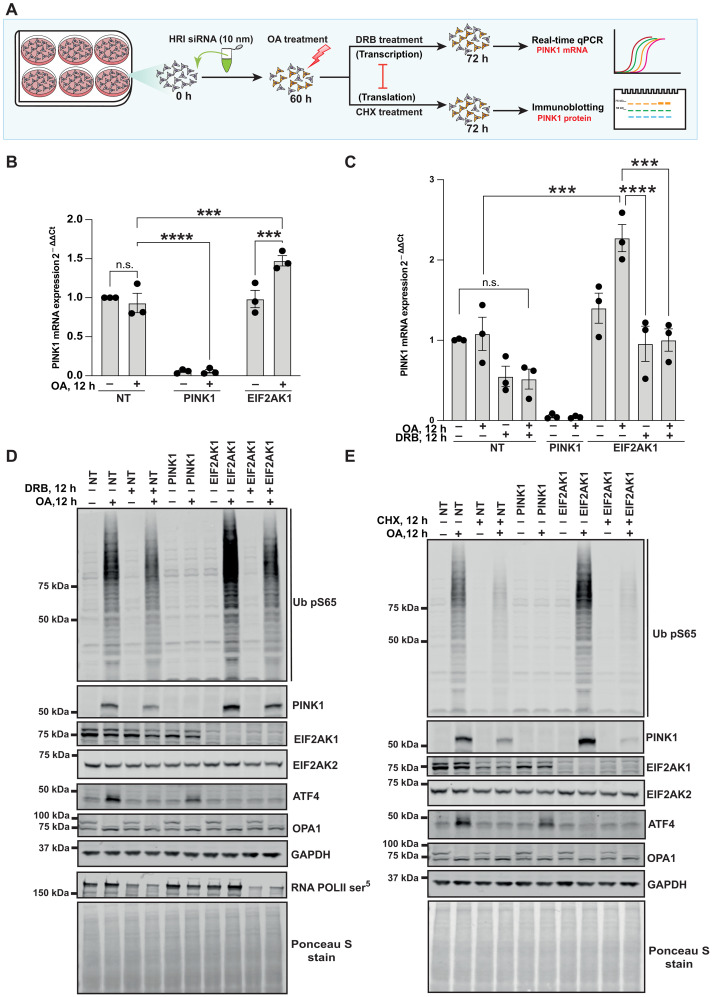
Transcriptional up-regulation of PINK1 in EIF2AK1 knockdown cells following mitochondrial depolarization. (**A**) Schematic of the siRNA workflow for studying transcriptional and translational regulation of PINK1. (**B**) HeLa cells either treated with non-targeting control siRNA pool (NT), siRNA pool targeting PINK1, or EIF2AK1for 72 hours were treated with OA in the last 12 hours. Relative mRNA levels of PINK1 measured by RT-PCR in HeLa cells with TBP used as a reference gene. (**C**) As in (B), relative mRNA levels of PINK1 measured by RT-PCR in HeLa cells with and without co-treatment of transcription inhibitor DRB, using TBP as a reference gene. (**D**) Corresponding immunoblot analysis for remaining two-thirds of the samples used for RT PCR analysis in (C). Immunoblot of Ub pS65, PINK1, EIF2AK1, EIF2AK2, ATF4, OPA1, GAPDH, RNA POLII phospho-S5 (DRB treatment control), and total protein as visualized by Ponceau S staining. (**E**) HeLa cells treated with non-targeting control siRNA pool (NT), siRNA pool targeting PINK1, or EIF2AK1for 72 hours were either treated with OA alone or co-treated with translation inhibitor CHX in the last 12 hours. Immunoblot of Ub pS65, PINK1, EIF2AK1, EIF2AK2, ATF4, OPA1, GAPDH, and total protein as visualized by Ponceau S staining. Data information: [(B) and (C)] All data are means ± SEM; statistical significance is displayed as ***P ≤ 0.001; ****P ≤ 0.0001; n.s., not significant. *n* = 3 biological replicates, (B) two-way ANOVA, Uncorrected Fisher’s LSD multiple comparisons test and (C) one-way ANOVA, Tukey’s multiple comparisons test. h, hours.

Previous studies have shown that, upon mitochondrial damage, PINK1 mRNA levels do not change and that PINK1 protein stabilization is prevented by pretreatment with the translation inhibitor, cycloheximide (CHX), suggesting a role for translation and new protein synthesis (*31*, *32*). Consistent with this, we performed a reverse transcription polymerase chain reaction (RT-PCR) time-course experiment following OA treatment in HeLa cells and found that PINK1 mRNA levels do not significantly change upon mitochondrial damage (fig. S11A). This contrasted with a significant time-dependent increase in ATF4 mRNA levels following OA treatment (fig. S11B). We next measured PINK1 mRNA levels under conditions of siRNA-mediated knockdown of EIF2AK1. Using human TBP as an internal control, our RT-PCR results revealed no change in PINK1 mRNA levels in EIF2AK1 knockdown cells under basal conditions; however, we observed a significant up-regulation of PINK1 mRNA in EIF2AK1 knockdown cells following OA-induced mitochondrial depolarization (Fig. 4B). Consistent with this, co-treatment of cells with the transcriptional inhibitor, 5,6-dichlorobenzimidazole 1-β-d-ribofuranoside (DRB), completely prevented PINK1 mRNA up-regulation in OA-induced EIF2AK1 knockdown cells with only mild impact on basal PINK1 mRNA levels (Fig. 4B). The specificity of our RT-PCR assay was confirmed by complete loss of PINK1 mRNA in cells transfected with PINK1 siRNA (Fig. 4B). Further, under these RT-PCR assay conditions, the efficiency and the specificity of EIF2AK1 knockdown were confirmed by prevention of OA-induced ATF4 mRNA up-regulation (fig. S11C) and complete abolishment of EIF2AK1 but not EIF2AK2 mRNA levels consistent with previous reports (fig. S11, D and E). In parallel, we assessed the impact of DRB co-treatment at the protein level in HeLa cells following EIF2AK1 knockdown, and, in agreement with the RT-PCR analysis, DRB prevented the OA-induced increase in PINK1 stabilization and activation with only modest effects on PINK1 in non-targeting siRNA–treated cells (Fig. 4D). We also observed similar results at the protein level using the transcriptional inhibitor, α-amanitin (fig. S11F). Furthermore, stabilization of PINK1 in EIF2AK1-silenced cells was inhibited by CHX treatment to a similar extent as non-targeting siRNA–treated cells following OA treatment, confirming the role of protein translation (Fig. 4, A and E, and fig. S12, A and B). Overall, these studies demonstrate that EIF2AK1 knockdown mediates transcriptional up-regulation and translation of PINK1 mRNA, and, in future work, it will be interesting to uncover the transcriptional mechanism and how this mRNA undergoes translation on sites of mitochondrial damage.

### Chemical inhibition of the ISR by ISRIB up-regulates PINK1 stabilization and activation in response to mitochondrial damage

**Fig. 5.**
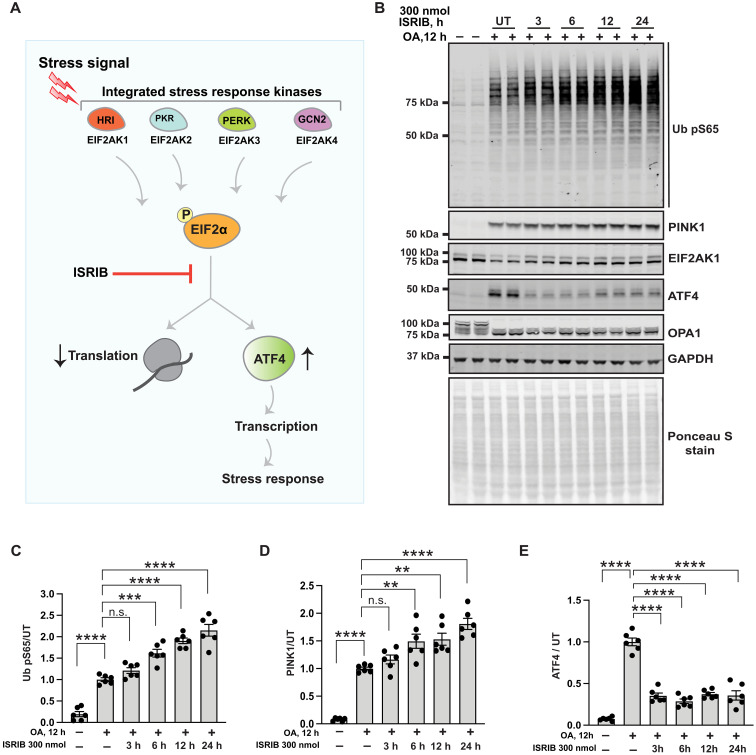
Chemical inhibition of the ISR by ISRIB enhances PINK1 stabilization and activation. (**A**) Schematic depicting the two outputs of the ISR: reduction in bulk protein synthesis and translational induction of ATF4 and its target genes. ISRIB reverses these effects of the ISR by acting downstream of the ISR kinases phosphorylation of eIF2α. (**B**) HeLa cells seeded in a 10-cm dish were co-treated with 300 nmol of ISRIB for 3, 6, 12, and 24 hours and OA for 12 hours or OA alone for 12 hours. Representative immunoblot of Ub pS65, PINK1, EIF2AK1, OPA1, GAPDH, ATF4, and total protein as visualized by Ponceau S staining. (**C** to **E**) Quantification for Ub pS65/GAPDH, PINK1/GAPDH, and ATF4/GAPDH ratio for (B), normalized to the ratio in untreated (UT) (+OA) samples using the LICOR Image Studio software. Data information: [(C) to (E)] All data are means ± SEM; statistical significance is displayed as **P ≤ 0.01; ***P ≤ 0.001; ****P ≤ 0.0001; n.s., not significant. *n* = 6 technical replicates (three biological replicates), one-way ANOVA, Tukey’s multiple comparisons test. h, hours.

The drug-like small-molecule ISR inhibitor (ISRIB) has been shown to antagonize eIF2α phosphorylation, leading to inhibition of the ISR via promotion of translation and inhibition of ATF4 expression (Fig. 5A) (*33*, *34*). We therefore investigated the impact of ISRIB on mitochondrial damage–induced activation of PINK1 signaling. We initially undertook a time course of 300 nM ISRIB in HeLa cells (3, 6, 12, and 24 hours) cotreated with OA for 12 hours. This revealed that ISRIB significantly enhanced stabilization and activation of PINK1 within 6 hours as measured by Ub pS65, and the effect of ISRIB was sustained up to 24 hours (Fig. 5, B to D). This was associated with ISRIB-induced inhibition of ATF4 following mitochondrial damage induced by OA (Fig. 5, B and E). Furthermore, treatment with ISRIB alone did not affect PINK1 signaling in healthy cells, indicating that ISRIB’s effects are specific to cells undergoing mitochondrial stress and ISR pathway activation (fig. S13A). Consistent with these results in HeLa cells, we also treated ARPE-19 cells with ISRIB and observed an increase in total PINK1 levels and Ub pS65 following OA-induced mitochondrial damage compared to those in non-ISRIB–treated cells (fig. S13B). Overall, these chemical studies with ISRIB complement the above genetic findings, suggesting ISRIB-like small molecules as a potential therapeutic strategy to enhance PINK1 mitophagy.

### Genetic or chemical inhibition of ISR enhances PINK1-dependent mitophagy

**Fig. 6.**
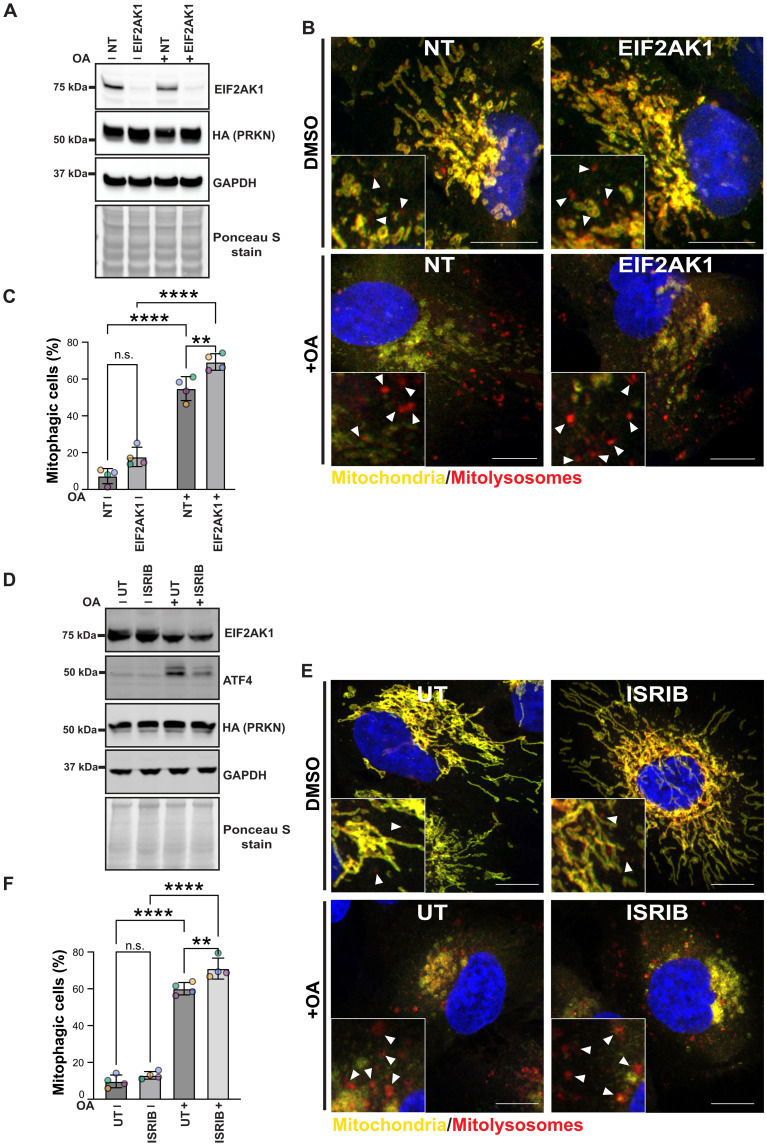
Genetic and chemical inhibition of EIF2AK1-mediated ISR enhances PINK1-Parkin–dependent mitophagy. (**A** and **B**) Representative immunoblots (A) or confocal images (B) of ARPE-19 cells stably expressing the mito-QC reporter and HA-Parkin, transfected with non-targeting siRNA (NT) or siRNA targeting EIF2AK1. Three days post-transfection, cells were treated with OA for 2 hours prior to analysis. (**C**) Quantification of mitophagy shown in (B) from four independent experiments (*n* = 4 biological replicates with >66 cells per replicate). (**D** and **E**) Representative immunoblots (D) or confocal images (E) of ARPE-19 cells stably expressing the mito-QC reporter and HA-Parkin, pretreated for 12 hours with 300 nM ISRIB and treated with OA for 2 hours prior analysis. (**F**) Quantification of mitophagy shown in (E) from four independent experiments (*n* = 4 biological replicates with >101 cells per replicate). (C) Two-way ANOVA, Tukey’s multiple comparisons test; and (F) one-way ANOVA, Tukey’s multiple comparisons test. Data information: Enlarged views are shown in the bottom corners, and arrowheads indicate examples of mitolysosomes. Nuclei were stained in blue (Hoechst). Scale bars, 10 μm. Overall data are means ± SD; statistical significance is displayed as **P ≤ 0.01; ****P ≤ 0.0001; n.s., not significant. DMSO, dimethyl sulfoxide.

We next determined whether the genetic knockdown of EIF2AK1 or chemical inhibition of ISR by ISRIB had an impact on PINK1-Parkin–dependent mitophagy. To monitor mitophagy, we used the previously established and well-characterized mito-QC assay in Parkin overexpressing ARPE-19 cells (*22*, *23*, *35*). This assay, using a stably expressed tandem mCherry–green fluorescent protein (GFP) tag attached to an OMM localization peptide (derived from the protein FIS1), relies on a fluorescent color change that occurs when mitochondria are delivered to lysosomes (referred to as mitolysosomes). Lysosomal acidity is sufficient to quench GFP, but not mCherry, and “red-only” mitolysosomes appear that can be easily quantified as a mitophagy readout. We observed that, under basal conditions, siRNA knockdown of EIF2AK1 did not significantly increase mitophagy compared to that of NT-control ARPE-19 Parkin–overexpressing cells (Fig. 6, A to C). In contrast, loss of EIF2K1 resulted in a significant increase in OA-induced mitolysosomes, indicative of increased mitophagy that is consistent with previous analyses of PINK1 activation (Fig. 6, A to C). We next co-treated ARPE-19 Parkin–overexpressing cells with or without OA and ISRIB. As with analysis of PINK1 signaling (fig. S13), we did not observe any significant effect of ISRIB on basal mitophagy (Fig. 6, D to F). However, we observed a small but significant mitophagy increase in cells co-treated with OA and ISRIB compared to those treated with OA alone (Fig. 6, D to F).

It was recently reported that EIF2AK1/HRI is a positive regulator of both PINK1-independent DFP-mediated mitophagy and PINK1-Parkin–dependent OA-mediated mitophagy (*36*). DFP is an iron chelator that mimics hypoxic conditions through stabilization of the oxygen-sensitive transcription factor HIF1α (hypoxia-inducible factor 1α) and has been described as the most potent mitophagy inducer in a PINK1-Parkin–independent manner (*37*). We performed siRNA-mediated knockdown of EIF2AK1 in mito-QC–expressing ARPE-19 cells treated with or without DFP in the presence or absence of bafilomycin A (BafA), a lysosomal inhibitor. Following DFP treatment, we observed significant reduction in the mitochondrial proteins HSP60, OMI, and COXIV in NT-targeted control cells that were prevented by co-treatment with BafA, indicating DFP-induced mitophagy (fig. S14, A and B). However, in EIF2AK1-depleted cells, mitochondrial proteins were similarly degraded upon DFP treatment, and no significant difference was observed with the NT-targeted cells, demonstrating that mitophagy still occurs in EIF2AK1 knockdown cells (fig. S14, A and B). To validate these findings, we measured mitophagy directly using fluorescence-activated cell sorting (FACS)–based assay of the mito-QC reporter and did not detect any significant difference in mitophagy between EIF2AK1 knockdown and NT-targeted cells under our assay conditions (fig. S14, C and D). Overall, our mitophagy analyses indicate that knockdown of EIF2AK1 or addition of ISRIB enhances PINK1 mitophagy and that the ISR pathway acts as a specific negative regulator of OA-induced mitophagy but does not affect the DFP-induced mitophagy pathway.

## DISCUSSION

**Fig. 7.**
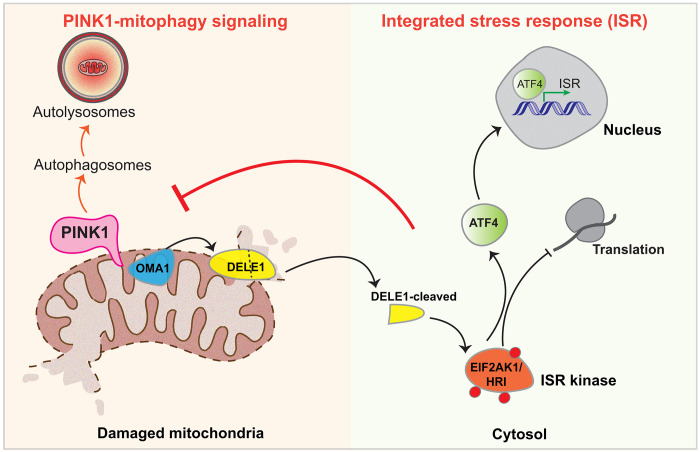
Model depicting negative interplay of DELE1-EIF2AK1/HRI ISR pathway and PINK1 mitophagy signaling. Upon mitochondrial stress both PINK1 and OMA1 become activated. OMA1 cleaves the inner mitochondrial membrane protein DELE1 to produce a C-terminal fragment (DELE1-cleaved). This accumulates in the cytosol, where it interacts with and activates the ISR kinase EIF2AK1/HRI. EIF2AK1 activation leads to a general reduction in protein translation while allowing the translation of specific mRNAs including the transcription factor ATF4. This leads to inhibition of PINK1 stabilization and mitophagy.

We have identified via an unbiased siRNA whole Ser/Thr kinome screen that the ISR kinase EIF2AK1/HRI acts as a negative regulator of PINK1-dependent signaling in cells. We show that, under conditions of mitochondrial stress, genetic knockdown of EIF2AK1 or its stress-induced activator DELE1 inhibits the ISR, leading to enhancement of PINK1 stabilization and activation. Furthermore, we have discovered that the small-molecule ISRIB, a potent inhibitor of the ISR, (via antagonizing the effects of eIF2α phosphorylation) enhances PINK1 activation and signaling. The physiological relevance of our findings is underscored by the demonstration that knockdown of EIF2AK1 or ISRIB stimulate PINK1-Parkin–dependent mitophagy induced by mitochondrial depolarization. Overall, our findings elaborate a model whereby the DELE1-EIF2AK1 relay pathway negatively interplays with the PINK1-Parkin mitophagy pathway (Fig. 7).

Mitochondrial stress–induced activation of EIF2AK1 by DELE1 leads to phosphorylation of eIF2α, resulting in the induction of the transcription factor ATF4 and global inhibition of mRNA translation and protein synthesis that mediate the ISR (*18*, *19*). In agreement with this, we observed time-dependent up-regulation of both ATF4 mRNA and protein levels following mitochondrial depolarization and that was significantly reduced by either EIF2AK1 or DELE1 genetic knockdown. Initially identified as a transcriptional repressor of the cyclic adenosine 3′,5′-monophosphate element (CRE), ATF4 can act as both a transcriptional activator and inhibitor in response to various stressors and is a critical downstream effector modulating transcription of multiple genes including those regulating mitochondrial function and quality control (*14*–*17*, *27*, *28*). The specific gene expression regulated by ATF4 has been attributed to the combinations of several factors such as ATF4 heterodimerization partners and posttranslational and histone modifications surrounding target genes (*14*–*17*, *27*, *28*). Previous studies indicated that PINK1 mRNA levels remain unchanged with mitochondrial stress, while the protein stabilizes rapidly unless pre-treated with the translation inhibitor CHX (*31*, *32*). Our results reveal that PINK1 mRNA transcription and translation is increased following mitochondrial depolarization under conditions of EIF2AK1 knockdown and suggests that the ISR perhaps, via ATF4, may repress PINK1 transcription. The PINK1 gene promoter lacks typical TATA and CAAT boxes but contains several putative and canonical regulatory sites for the transcription factors AP1F, nuclear factor κB (NF-κB), and CREB (*38*, *39*). A previous study also found that PINK1-Parkin mitophagy was increased in response to mitochondrial misfolding stress in ATF4 KO cells (*29*). However, how PINK1 is transcriptionally regulated by any stress is largely unexplored in the field, and, in future work, it would be interesting to investigate the mechanism by which PINK1 mRNA is transcriptionally regulated by ATF4 under basal and mitochondrial stress conditions. Furthermore, mitochondrial proteins have been shown to be actively translated on the mitochondrial surface, and cryo–electron tomography–visualized ribosomes have been observed interacting with the TOM complex in both basal and mitochondrial depolarization states, facilitating co-translational import of select mitochondrial proteins although the effect of ISR stress in this context is not known (*40*, *41*).

In a recent CRISPRi screen in human K562 cells, EIF2AK1/HRI was identified as a positive regulator of DFP-induced mitophagy (*36*). The same group also reported that EIF2AK1 acted as a positive regulator for PINK1-Parkin mitophagy in HeLa cells that is contrary to our findings presented here (*36*). At present, we are unable to reconcile the differences between the studies although, in our study, we have been able to demonstrate that EIF2AK1 acts as a negative regulator of PINK1 mitophagic signaling in multiple human cell lines including HeLa, ARPE-19, SK-OV-3, and U2OS cells. Furthermore, under our assay conditions, the small-molecule ISRIB also enhanced PINK1 stabilization, activation, and mitophagy in a similar way to what we observed with genetic knockdown of EIF2AK1, supporting a role for the ISR in negatively regulating PINK1 signaling following mitochondrial stress. The relatively smaller effect observed for EIF2AK1 knockdown or ISRIB on OA-induced mitophagy compared to OA-induced PINK1 activity signaling likely reflects the high level of mitophagy conferred by Parkin overexpression in this system via Parkin-mediated feed-forward amplification of downstream signaling.

In future studies, it would be interesting to determine whether EIF2AK1 regulates PINK1 in cells more relevant to PD including human iNeurons and primary mouse neurons that exhibit robust PINK1-Parkin–dependent expression and signaling (*42*–*44*). It has been reported in neurons that mRNA is cotransported with mitochondria along axons and locally translated to enable mitophagy in distal parts of the axon (*45*), and it would be interesting to investigate whether the levels of PINK1 mRNA in axons of neurons are regulated by ISR pathway activation induced by mitochondrial stress. It would also be important to evaluate whether EIF2AK1 and ISR regulate endogenous PINK1-Parkin–dependent signaling and mitophagy in vivo. Using the mito-QC mouse reporter line, it has recently been reported that skeletal hindlimb muscle represents the tissue of choice for studying PINK1-dependent mitophagy in vivo with significant reduction of mitophagy in PINK1 KO mice (*46*). EIF2AK1 KO mice have been reported to be viable and display mild alterations in erythrocytes but no gross morphological abnormalities in other tissues (*26*). In future work, it would be interesting to cross EIF2AK1 KO mice with the mito-QC mouse reporter line and determine whether EIF2AK1 loss leads to enhanced PINK1 mitophagy in skeletal muscle. It would also be interesting to treat WT and PINK1 KO mice with ISRIB or analogs to assess whether these can enhance PINK1-dependent mitophagy in skeletal muscle in vivo.

The original DELE1-HRI discovery studies demonstrated in HeLa cells that the mitochondrial protease OMA1 was critical for the cleavage of DELE1 with concomitant release of the C-terminal fragment of DELE1 into the cytosol (*18*, *19*). However, a recent study has reported that the mitochondrial protease HtrA2 can also mediate the cleavage of DELE1 (*47*). Additionally, a recent study highlighted the critical role of the SIFI complex, an E3 ligase that degrades both the active form of EIF2AK1 and the cleaved DELE1 fragment along with accumulated unimported mitochondrial proteins during mitochondrial import stress (*30*). This degradation process is vital for silencing the ISR and may aid in restoring TOM import and cellular equilibrium after stress. However, a beneficial role for ISR activation has been suggested from studies of mitochondrial misfolding stress that showed that down-regulation of the ISR and mitochondrial UPR response (by KO of ATF transcription factors) resulted in substantial mitochondrial dysfunction and proteostasis despite cells being able to continue mitophagy (*29*). These data suggest that the ISR-mediated inhibition of mitophagy may enable cells to attempt to repair damaged mitochondria via ISR transcriptional responses involving chaperones and proteases and that mitophagy represents a final safety mechanism if the repair is unsuccessful. In our experiments, we also observed a reduction in endogenous EIF2AK1 levels following OA-induced mitochondrial depolarization, suggesting the involvement of the SIFI complex in this response. In future studies, it would be interesting to determine the role of the ISR components and their interplay with mitophagy in cells that are physiologically relevant to PD including neurons using diverse mitochondrial stressors to understand the context in which ISR inhibition may be beneficial in PD.

To date, the best-characterized negative regulator of PINK1/Parkin-induced mitophagy signaling is the deubiquitinase (DUB) USP30, discovered following an overexpression screen with a Flag-tagged human DUB cDNA library, with phase 1 human trials of USP30 inhibitors underway in patients with PD (Mission Therapeutics) (*48*). Our discovery that ISRIB enhances PINK1 stabilization, activation, and mitophagy opens up therapeutic approaches to treat PD. ISRIB has been demonstrated to restore translational inhibition induced by ISR activation and to prevent gene expression induced by ATF4 by acting downstream of phosphorylated eIF2α (*15*, *33*). In mammalian cells exposed to ER stress inducers such as thapsigargin and tunicamycin, ISRIB effectively blocked the PERK-mediated induction of ATF4 (*33*). ISRIB was found to confer neuroprotection in select animal models in vivo (*33*, *34*) and was recently reported to rescue neurodegeneration of amyotrophic lateral sclerosis (ALS) gene *VAPB*-iPSC-derived motor neurons (*49*). Furthermore, two clinical drug analogs of ISRIB, ABBV-CLS-7262 (AbbVie/Calico) and DNL-343 (Denali Therapeutics), are now undergoing evaluation in clinical trials of ALS (*50*). Critically in our studies, ISRIB and EIF2AK1 knockdown did not affect PINK1 signaling in healthy basal cells and was specific to cells undergoing mitochondrial stress, suggesting that therapeutic approaches to target the ISR such as ISRIB or inhibition of EIF2AK1 activation by DELE1 may be associated with fewer side effects.

Overall, our current analysis strongly indicates that mitochondrial stress–induced activation of the ISR, mediated by the EIF2AK1/HRI kinase, is a negative regulator of PINK-dependent mitophagic signaling. Further investigation in PD models will be needed to determine whether inhibition of ISR signaling represents a new potential therapeutic strategy against PD.

## MATERIALS AND METHODS

### Materials

cDNA constructs for mammalian tissue culture were amplified in *E. coli* DH5α cells and purified using a NucleoBond Xtra Midi kit (no. 740410.50, Macherey-Nagel). All DNA constructs were verified by DNA sequencing, which was performed by the Sequencing Service, School of Life Sciences, University of Dundee, using DYEnamic ET terminator chemistry (Amersham Biosciences) on Applied Biosystems automated DNA sequencers. DNA for bacterial protein expression was transformed into *E. coli* BL21 DE3 RIL (codon plus) cells (Stratagene). All cDNA plasmids, CRISPR guide RNAs, antibodies, and recombinant proteins generated for this study are available via our reagents website: https://mrcppureagents.dundee.ac.uk/.

### Reagents

Antimycin A (A8674), oligomycin (75351), CHX (C7698), DRB (D1916), ISRIB (SML0843), and DFP (379409) were purchased from Sigma-Aldrich. α-Amanitin (4025) was purchased from Tocris/Bio-Techne. Bafilomycin A1 adenosine triphosphatase inhibitor (BafA) (BML-CM110) was purchased from Enzo Life Sciences.

### Antibodies for biochemical studies

The following primary antibodies were used: Ub phospho-Ser^65^ [Cell Signaling Technology (CST), catalog no. 62802)], OPA1 (BD Biosciences, catalog no. 612607), Rab8A (Sigma-Aldrich, catalog no. WH0004218M2), Rab8A (Abcam, catalog no. Ab241061), Rab8A phospho-S111 (Abcam, catalog no. Ab267493), PINK1 (Novus, catalog no. BC100-494; CST, catalog no. 6946 T), PINK1 (in-house generated by Dundee Cell Products), GAPDH (Santa Cruz Biotechnology, catalog no. sc-32233), EIF2AK2 (Abcam, catalog no. Ab184257), EIF2AK3 (Abcam, catalog no. Ab229912), EIF2AK4 (Abcam, catalog no. Ab134053), ATF4 (CST, catalog no. 11815S), OMA1 (Santa Cruz Biotechnology, catalog no. sc-515788), TRIM28 (anti-KAP1; Abcam, catalog no. Ab109287), RPS6KB2 (Abcam, catalog no. Ab184551), BCR (Abcam, catalog no. Ab233709), BRD2 (Abcam, catalog no. Ab139690), MAP3K7 (Abcam, catalog no. Ab109526), CDC7 (Abcam, catalog no. Ab229187), ROCK1 (Abcam, catalog no. Ab134181), EEF2K (Abcam, catalog no. Ab45168), CAMK1D (Abcam, catalog no. Ab172618), GRK2 (Abcam, catalog no. Ab227825), CNSK2A1 (Abcam, catalog no. Ab70774), CSNK1A1 (Abcam, catalog no. Ab206652), GSK3B (Abcam, catalog no. Ab32391), MAPK3 (Abcam, catalog no. Ab32537), PBK/SPK (Abcam, catalog no. Ab236872), MAP3K5 (Abcam, catalog no. Ab45178), MAP2K2 (Abcam, catalog no. Ab32517), MAP2K6 (Abcam, catalog no. Ab33866), MAP2K7 (Abcam, catalog no. Ab52618), hemagglutinin (HA) (Invitrogen, catalog no. 26183), HA (Sigma-Aldrich, catalog no. 11867423001), RNA polymerase II CTD repeat YSPTSPS (phospho-S5) (Abcam, catalog no. Ab817), HIF1α (R&D Systems, catalog no. MAB1536), HSP60 (CST, catalog no. 4870S), COXIV (CST, catalog no. 4850S), anti-LC3 A/B (CST, catalog no. 4108S), and vinculin (Abcam, catalog no. Ab129002). The following polyclonal antibodies were produced by the MRCPPU Reagents and Services at the University of Dundee in sheep: EIF2AK1 (DA219) and OMI (S802C).

### siRNA screens and follow-up experiments

The siRNA screens were performed using a human siRNA library (Horizon Dharmacon) designed to target 428 Ser/Thr kinases. The list of target siRNA pools and their oligonucleotide sequences are listed in table S2. HeLa cells (1.5 ml) were seeded in six-well plates at 37,000 cells/ml and transfected using 10 nm of siRNA and 1.5 μl of the Lipofectamine RNAiMAX transfection Reagent (Thermo Fisher Scientific) per well. Cells were cultured for 52 hours after which were stimulated with oligomycin (1 μM) and antimycin (10 μM) (OA). After further 20 hours, cells were lysed in 50 μl of lysis buffer, centrifuged at 17,000*g* for 15 min at 4°C, quantified by Bradford Assay (Thermo Fisher Scientific), and subjected to immunoblot analysis. Each siRNA screening experiment also included untreated cells, positive control PINK1 targeting siRNA, and a negative control siRNA [non-targeting siRNA (NT)]. The siRNA studies for further validation of EIF2AK1 with independent pooled siRNA for EIF2AK1, EIF2AK2, EIF2AK3, and EIF2AK4 as well as single siRNAs for EIF2AK1 (Dharmacon; table S2) were also performed in four different cell lines (HeLa, SKOV3, U2OS, and ARPE-19) as above. However, in the further validation studies, the cells were cultured for 60 hours and then stimulated with oligomycin (1 μM) and antimycin (10 μM) (OA). After further 12 hours, cells were lysed in 50 μl of lysis buffer, centrifuged at 17,000*g* for 15 min at 4°C, quantified by the Coomassie (Bradford) Protein Assay Kit (Thermo Fisher Scientific), and subjected to immunoblot analysis. The siRNA studies for targeting DELE1 (Horizon Dharmacon; table S2) alongside EIF2AK1 were also performed in a similar manner.

### Generation of EIF2AK1 CRISPR-Cas9 KO cells

A full transcript map of the *EIF2AK1* locus was constructed by combining data from both National Center for Biotechnology Information (NC_000007.14 (6022247.6059175, complement) and Ensembl (ENSG00000086232). KO guides were chosen to target as far upstream as possible within an exon common to all published and predicted variants to ensure complete disruption of all possible transcripts following CRISPR targeting. Three pairs of CRISPR-Cas9 guides were designed: a pair targeting exon 2, G1 single-guide RNA targeting exon 2, and G2 single-guide RNA targeting exon 2 of the *EIF2AK1* gene, and this would be predicted to abolish expression of full-length EIF2AK1 protein (fig. S9, A to D). Complementary oligonucleotides were designed and annealed to generate double-stranded DNA inserts with overhangs compatible to Bbs I–digested destination vectors. The sense guide insert was subsequently cloned into Bbs I–digested pBABED P U6 (DU48788, University of Dundee) and the antisense cloned into Bbs I–digested pX335 (Addgene, no. 42335), yielding clones DU69746 and DU69747, respectively.

HeLa cells were co-transfected with 1 μg of each plasmid using PEI in a 10-cm dish. Following transfection, cells were allowed to recover for 24 hours before addition of puromycin selection (1 μg/ml) for a further 48 hours. The cell pool was subsequently analyzed for EIF2AK1 depletion by immunoblotting then single-cell sorted via FACS. Following recovery, individual clones were analyzed for EIF2AK1 depletion by immunoblotting (fig. S9E) and the promising clones A2 and A3 further verified by PCR and shotgun cloning and sequencing. Briefly, genomic DNA was isolated, and a 1.8-kb region containing exon 2 was amplified by PCR (forward primer: CACGGCATCTTTCTGCTGATCC; reverse primer: TCCAATTTTTGTATACCAG ACGCTTTCC) (fig. S9, B and C). The resulting PCR products were subcloned into the holding vector pSC-B (StrataClone Blunt PCR Cloning Kit, Agilent Technologies) and 16 colonies (white) picked for each of the clonal lines. Plasmid DNAs were isolated and cut with Eco RI to verify insert size and 14 positive samples for each line sent for sequencing with primers M13F and M13R. Indel formation often leads to a wide range of variations between targeted alleles, leading to a variety of variations between targeted alleles; thus, direct sequencing of amplified amplicon pools yields poor-quality data around the CRISPR target site(s). We have found in practice that analysis of >8 to 10 shotgun clones from a given clonal line is sufficient to verify the allelic population with precision, for hyper- and hypotriploid lines more sequencing reads may be required to be confident that all alleles are accounted for. Sequencing of the A2 and A3 clones showed three copies of the chromosome in each case, and each indel was confirmed to lead to frameshift and premature termination of EIF2AK1, further corroborating the Western data and confirming successful KO.

### Cell culture and transfection

HeLa, U2OS, and SK-OV-3 cells were routinely cultured in standard Dulbecco’s modified Eagle’s medium (DMEM) supplemented with 10% (v/v) fetal bovine serum (FBS), 2 mM l-glutamine, penicillin (100 U/ml), and streptomycin (0.1 mg/ml). ARPE-19 cells (American Type Culture Collection, CRL-2302) were routinely cultured in 1:1 DMEM:F-12 medium supplemented with 10% (v/v) FBS, 2 mM l-glutamine, penicillin (100 U/ml), and streptomycin (0.1 mg/ml). The cells were passaged by washing the cells (80 to 90% confluency) with phosphate-buffered saline (PBS) followed by incubation with trypsin/EDTA. To inactivate trypsin/EDTA (1:1), pre-warmed cell culture medium was added after 5 min, and cells were centrifugated at 1200 rpm for 3 min. The cell number in the suspension was counted by an automated cell counter and seeded into new culture dishes at required densities. All cells were cultured at 37°C and 5% CO_2_ in a humidified incubator and routinely tested for mycoplasma. Where indicated, cells were treated with oligomycin (1 μM)/antimycin A (10 μM) (OA) for mitochondrial depolarization and 300 nM ISRIB as specified for chemical inhibition of the ISR. For iron chelation, DFP was used at 1 mM concentration, and inhibition of lysosomal degradation was achieved by BafA treatment at 50 nM. Inhibition of transcription was achieved by treatment with DRB at 50 μM, α-amanitin at 5 μg/ml and translation by treatment with CHX at 1 μg/ml for 12 hours.

### Whole-cell lysate preparation

Cells were lysed in an ice-cold lysis buffer containing 50 mM tris-HCl (pH 7.45), 150 mM NaCl, 1% (by volume) Triton X-100, 5 mM MgCl_2_, 1 mM sodium orthovanadate, 50 mM NaF, 10 mM 2-glycerophosphate, 5 mM sodium pyrophosphate, microcystin LR (0.5 μg/ml; Enzo), and cOmplete EDTA-free protease inhibitor cocktail (Roche) with freshly added 1× phosphatase inhibitor cocktail (Sigma-Aldrich) and Benzonase (2 μl/ml; Sigma-Aldrich). Lysates were clarified by centrifugation at 17,000*g* at 4°C for 15 min, and supernatants were quantified by Bradford assay (Thermo Fisher Scientific) and bicinchoninic acid assay (Thermo Fisher Scientific).

### Immunoblotting

Samples were subjected to SDS–polyacrylamide gel electrophoresis (12% tris-glycine gels and 4 to 12% bis-tris gels; Novex) and transferred onto nitrocellulose membranes. Membranes were blocked for 1 hour at room temperature with 5% nonfat milk or 5% bovine serum albumin (BSA) in tris-buffered saline [TBST; 50 mM tris-HCl and 150 mM NaCl (pH 7.5)] containing 0.1% Tween 20 and incubated at 4°C overnight with the indicated antibodies, diluted in 5% BSA or 5% nonfat milk. Highly cross-absorbed H+L secondary antibodies (Life Technologies) conjugated to (IRDye 800CW or IRDye 680RD Infrared Dyes) were used at 1:10,000 in TBST for 1 hour, and the membrane was washed once with TBS then imaged using the OdysseyClx Western blot imaging system.

### Protein purification in *E. coli*

Full-length WT EIF2AK1 was expressed in *E. coli* as N-terminal GST fusion protein and purified as described previously for GST proteins (*51*). Briefly, BL21 Codon+ transformed cells were grown at 37°C to an optical density at 600 nm (OD_600_) of 0.3, then shifted to 16°C, and induced with 250 μM isopropyl-β-d-thiogalactopyranoside (IPTG) at OD_600_ of 0.5. Cells were induced with 250 μM IPTG at OD of 0.6 and were further grown at 16°C for 16 hours. Cells were pelleted at 4000 rpm and then lysed by sonication in lysis buffer containing 50 mM tris-HCl (pH 7.5), 150 mM NaCl, 0.1 mM EGTA, 0.1% (v/v) 2-mercaptoethanol, and 270 mM sucrose. Lysates were clarified by centrifugation at 30,000*g* for 30 min at 4°C followed by incubation with 1 ml of glutathione (reduced form) agarose resin for 1.5 hours at 4°C. The resin was washed thoroughly in wash buffer containing 50 mM tris-HCl (pH 7.5), 200 mM NaCl, 0.5 mM TCEP, and 10% glycerol, and the protein was eluted by incubation with wash buffer containing 10 mM glutathione for 1 hour at 5° to 7°C. The eluted supernatant was dialyzed against wash buffer at 5° to 7°C overnight and concentrated, and the final sample was flash frozen.

### Quantitative RT-PCR

HeLa cells seeded in 10-cm dishes were washed and collected in sterile PBS. Samples were divided such that two-thirds of cells were used for immunoblot analysis as described above and the remaining one-third were snap frozen for total RNA isolation. RNA was extracted for each sample using the PureLink RNA Mini Kit (Invitrogen by Thermo Fisher Scientific, no. 12183025). cDNA synthesis was achieved using the PrimeScriptRT reagent Kit with gDNA Eraser (Takara, no. RR047A) with 1 μg of RNA as template and following the manufacturer’s instructions. The obtained cDNA was used as a template for quantitative PCR (qPCR) with TB Green Premix Ex Taq II (Tli TNaseJ Plus) (Takara, no. RR820L) and the following primers (Sigma-Aldrich): PINK1: 5′-AGACGCTTGCAGGGCTTTC-3′ [forward (F)] and 5′-GGCAATGTAGGCATGGTGG-3′ [reverse (R)]; β-actin: 5′-AGAAGGATTCCTATGTGGGCG-3′ (F) and 5′-CATGTCGTCCCAGTTGGTGAC-3′ (R); TBP: 5′-TGTATCCACAGTGAATCTTGGTTG-3′ (F) and 5′-GGTTCGTGGCTCTCTTATCCTC-3′ (R);

ATF4: 5′-CCAACAACAGCAAGGAGGAT-3′ (F) and 5′-GGGGCAAAGAGATCACAAGT-3′ (R); HRI: 5′-ACCCCGAATATGACGAATCTGA-3′ (F) and 5′-CAAGTGCTCCAGCAAAGAAAC-3′ (R); and PKR: 5′-GCCGCTAAACTTGCATATCTTCA-3′ (F) and 5′-TCACACGTAGTAGCAAAAGAACC-3′ (R). Real-time qPCR was performed in triplicates on thermocycler CFX Opus 384 (Bio-Rad Laboratories) operated by CFX Maestro software (Bio-Rad) using cycling protocol of 30 s at 95°C followed by 40 cycles of 5 s at 95°C and 60 s at 60°C, and followed by melting curve from 65° to 95°C. The fold change in expression was calculated using the 2^−ΔΔCt^ method (*52*). Data were analyzed in Microsoft Excel and GraphPad Prism version 10.0.3 using one-way analysis of variance (ANOVA; multiple comparisons).

### Mitophagy assay

Cells stably expressing mito-QC mitophagy reporter system (mCherry-GFP-FIS1^101–152^) and HA-Parkin were seeded onto sterile glass coverslips in 24-well dishes. After treatment, coverslips were washed twice with PBS, fixed with 3.7% (w/v) formaldehyde and 200 mM Hepes (pH 7.0) for 10 min, and washed twice with PBS. After nucleus counterstaining with Hoechst-33258 dye (1 μg/ml), slides were washed and mounted in ProLong Gold (Invitrogen). For quantification, images were taken with Nikon Eclipse Ti wide-field microscope (Plan Apo Lambda 60× Oil Ph3 DM). All the images were processed with Fiji v1.52n software (ImageJ, National Institutes of Health). Quantification of mitophagy (red-only dots) was performed from four independent experiments counting more than 66 cells per condition. Images were processed with the mito-QC counter plugin as previously described (*22*). Note that we observed some variation between cells and replicates. To keep quantitation consistent, we set a threshold above which the cells were considered as mitophagic based on the untreated WT condition. As quantitation was automated, initial blinding to sample ID was not performed.

### Flow cytometry analysis

ARPE-19 cells were seeded in a 6-cm dish. After treatment, cells were washed with PBS, trypsinized for 5 min, and centrifuged 3 min at 1200 rpm. The pellet of cells was resuspended in 250 μl of PBS, and 2 ml of 3.7% (w/v) formaldehyde and 200 mM Hepes (pH 7.0) were added. After 30 min at room temperature, 3 ml of PBS was added before centrifugation 5 min at 1200 rpm. Last, the pellet of cells was resuspended in 1% fetal calf serum in PBS and directly analyzed by flow cytometry. For each independent experiment, at least 5 × 10^4^ cells were acquired on LSRFortessa cell analyzer. On the basis of forward-scatter and side-scatter profiles, living cells were gated. As negative control, cells expressing any mitophagy reporter can be used. To quantify the percentage of cells underdoing mitophagy, the ratio GFP/mCherry was analyzed. The gate used for the nontreated condition or control cells was applied to all the other conditions. The value used for this was based on quantitation of microscopy data from mito-QC cells that showed around 7% (mito-QC) of cells had red-only puncta above the value of the mean.

### Statistical analyses

All statistical analyses were performed in GraphPad Prism version 10.0.3. Representative results of at least two or three independent experiments (biological replicates) are shown in all panels as specified. For immunoblot quantifications, level of each protein was normalized to GAPDH or VINCULIN and expressed as fold change. Statistical significance was determined either by ordinary one-way ANOVA or by ordinary two-way ANOVA with the appropriate multiple correction test. *P* values are indicated as *P < 0.05, **P < 0.01, ***P < 0.001, and ****P < 0.0001. Not significant (n.s.), *P* > 0.05. The details of each statistical test, *n* numbers, and graph used are specified in the relative figure legends.

### HEK293 cell transfection

Human embryonic kidney (HEK) 293 cells were transiently transfected with 5 μg of each of C-terminal HA-tagged EIF2AK1 WT or kinase-inactive (KI) or WT EIF2AK4 plasmid for 24 hours using PEI in a 10-cm dish. Twenty-four hours post-transfection, cells were lysed and immunoblotted with EIF2AK1 and HA antibodies (fig. S5, B and C), and membranes were analyzed using the OdysseyClx Western blot imaging system.

### Data analysis

Raw values from LICOR analyzed immunoblots for individual replicate experiments are shown in relevant figures and the supplementary figures.
