# Supplementary material for: Kinome screening identifies integrated stress response kinase EIF2AK1/HRI as a negative regulator of PINK1 mitophagy signaling
Source: Sci Adv. 2025 May 9;11(19):eadn2528. doi: 10.1126/sciadv.adn2528 (PMC12063660; doi:10.1126/sciadv.adn2528)
Supplement: Supplementary file 1 — Figs. S1 to S14 Legends for tables S1 to S3 [file sciadv.adn2528_sm.pdf]

Science Advances
AAAS

Supplementary Materials for

Kinome screening identifies integrated stress response kinase EIF2AK1/HRI as a negative regulator of PINK1 mitophagy signaling

Pawan K. Singh *et al.*

Corresponding author: Shalini Agarwal, sagarwal001@dundee.ac.uk; Miratul M. K. Muqit, m.muqit@dundee.ac.uk

Sci. Adv. **11**, eadn2528 (2025)
DOI: 10.1126/sciadv.adn2528

The PDF file includes:

Figs. S1 to S14
Legends for tables S1 to S3

Other Supplementary Material for this manuscript includes the following:

Tables S1 to S3

Fig. S1.

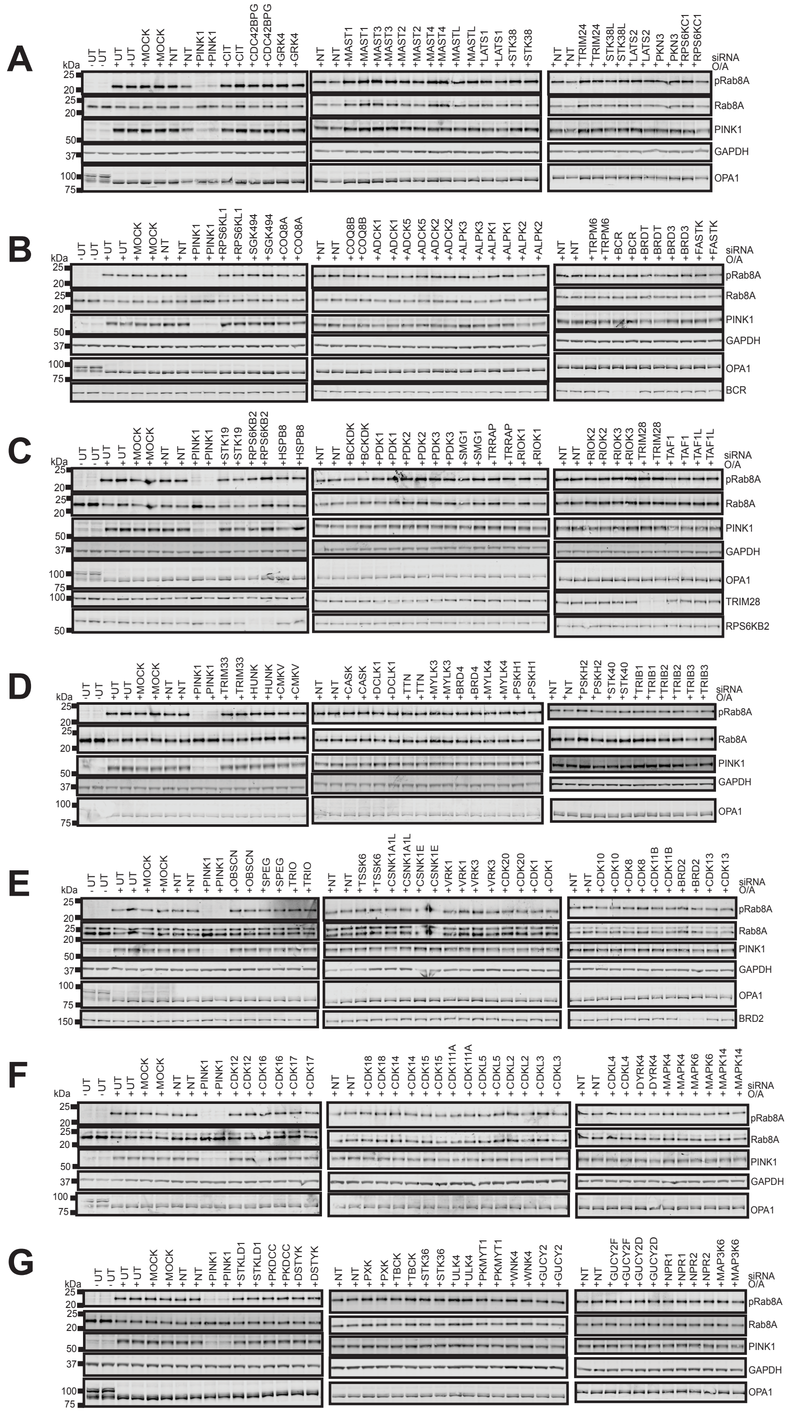

Immunoblot analysis of siRNA Ser/Thr kinome screen for regulators of PINK1-mediated Rab8A Ser111 phosphorylation. (**A-G**) HeLa cells transfected with the indicated siRNA pools of 108 set of Ser/Thr Kinases (Dharmacon) for 72 h and treated with OA in the last 20 h. Cells were lysed and immunoblotted for the indicated antibodies: Rab8A pS111, Rab8A, PINK1, GAPDH, OPA1, BCR, TRIM28, RPS6KB2, BRD2. Rabbit Rab8A pS111 and Mouse Rab8A antibodies were multiplexed on the same membrane throughout. All the immunoblots were developed using the LI-COR Odyssey CLx Western blot imaging system.

Fig. S2

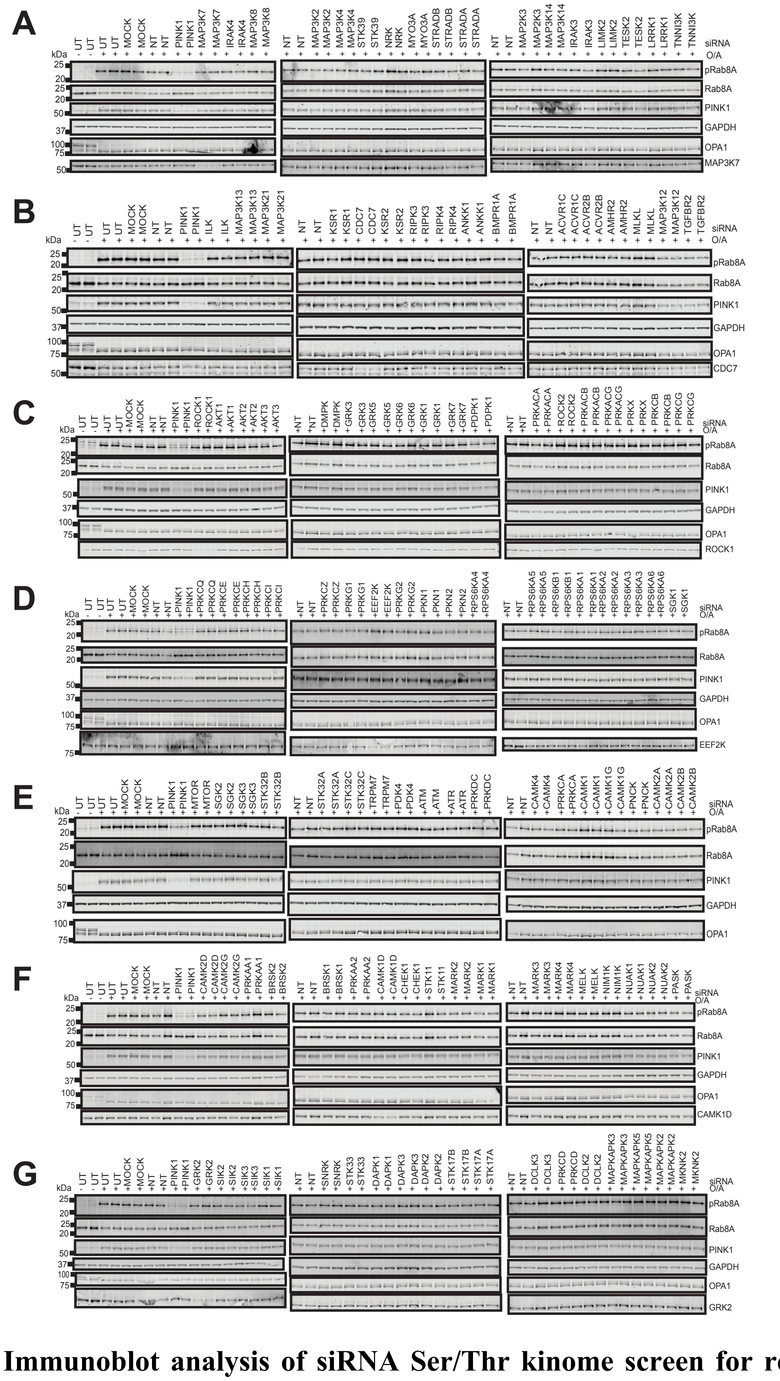

Immunoblot analysis of siRNA Ser/Thr kinome screen for regulators of PINK1-mediated Rab8A Ser111 phosphorylation. (**A-G**) HeLa cells transfected with the indicated siRNA pools of 123 set of Ser/Thr Kinases (Dharmacon) for 72 h and treated with OA in the last 20 h. Cells were lysed and immunoblotted for the indicated antibodies: Rab8A pS111, Rab8A, PINK1, GAPDH, OPA1, MAP3K7, CDC7, ROCK1, EEF2K, CAMK1D, GRK2. Rabbit Rab8A pS111 and Mouse Rab8A antibodies were multiplexed on the same membrane throughout. All the immunoblots were developed using the LI-COR Odyssey CLx Western blot imaging system.

Fig. S3.

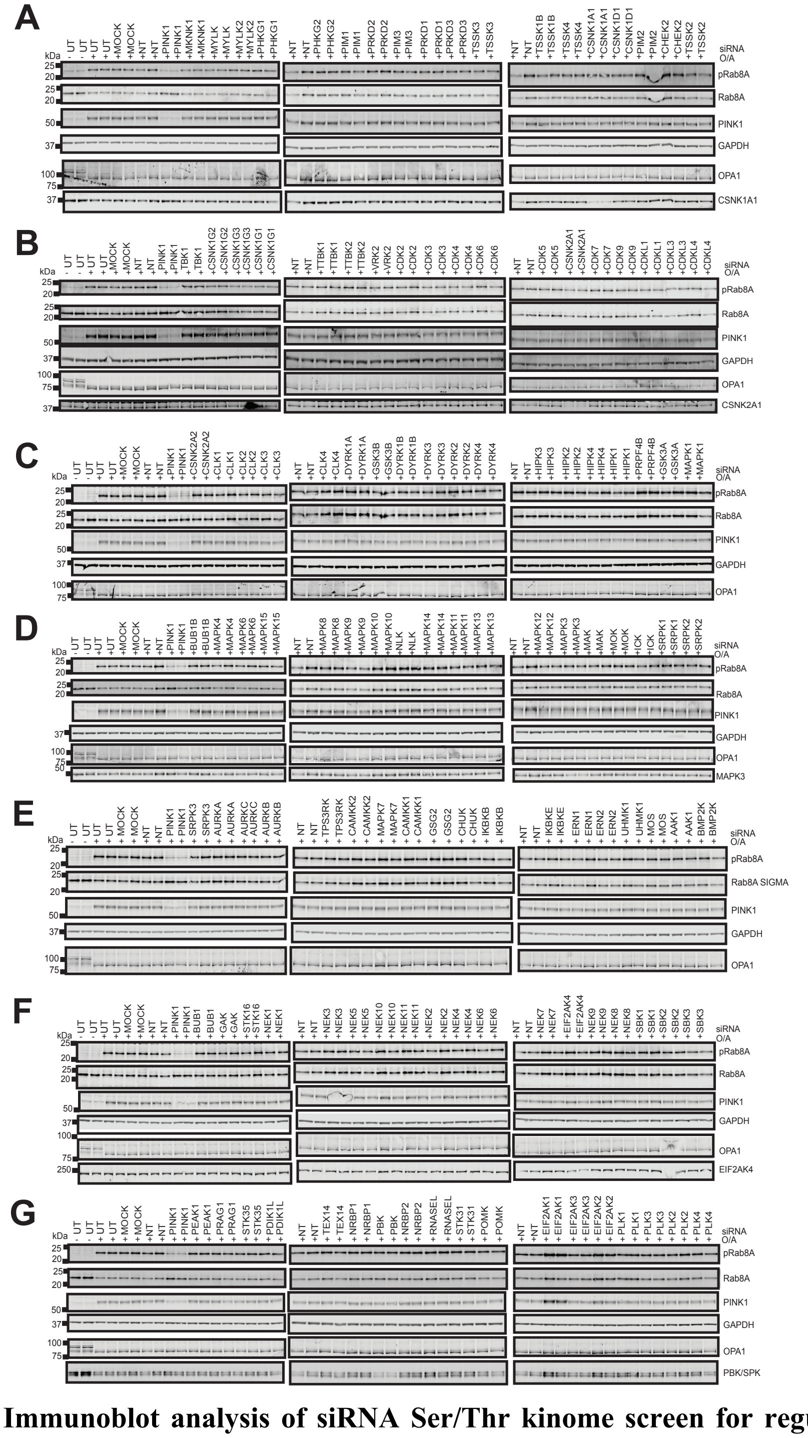

Immunoblot analysis of siRNA Ser/Thr kinome screen for regulators of PINK1-mediated Rab8A Ser111 phosphorylation. (**A-G**) HeLa cells transfected with the indicated siRNA pools of 126 set of Ser/Thr Kinases (Dharmacon) for 72 h and treated with OA in the last 20 h. Cells were lysed and immunoblotted for the indicated antibodies: Rab8A pS111, Rab8A, PINK1, GAPDH, OPA1, CSNK1A1, CSNK2A1, MAPK3, EIF2AK4, PBK/SPK. Rabbit Rab8A pS111 and Mouse Rab8A antibodies were multiplexed on the same membrane throughout. All the immunoblots were developed using the LI-COR Odyssey CLx Western blot imaging system.

Fig. S4.

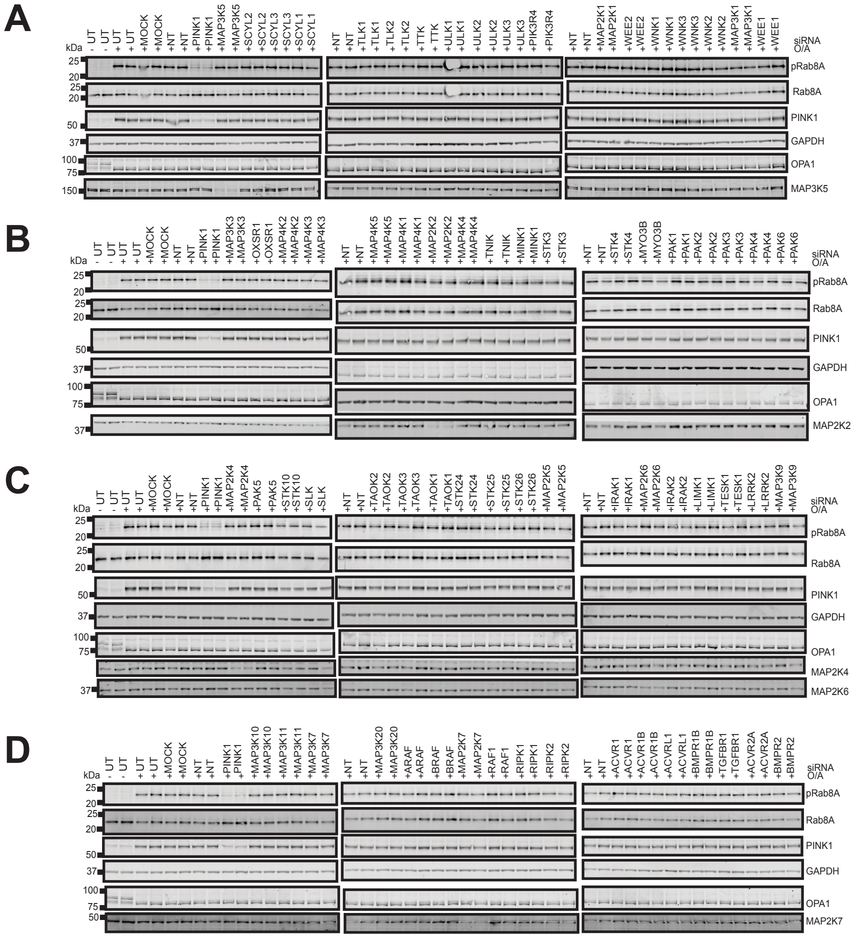

Immunoblot analysis of siRNA Ser/Thr kinome screen for regulators of PINK1-mediated Rab8A Ser111 phosphorylation. (**A-G**) HeLa cells transfected with the indicated siRNA pools of 71 set of Ser/Thr Kinases (Dharmacon) for 72 h and treated with OA in the last 20 h. Cells were lysed and immunoblotted for the indicated antibodies: Rab8A pS111, Rab8A, PINK1, GAPDH, OPA1, MAP3K5, MAP2K2, MAP2K6, MAP2K7. Rabbit Rab8A pS111 and Mouse Rab8A antibodies were multiplexed on the same membrane throughout. All the immunoblots were developed using the LI-COR Odyssey CLx Western blot imaging system.

Fig. S5.

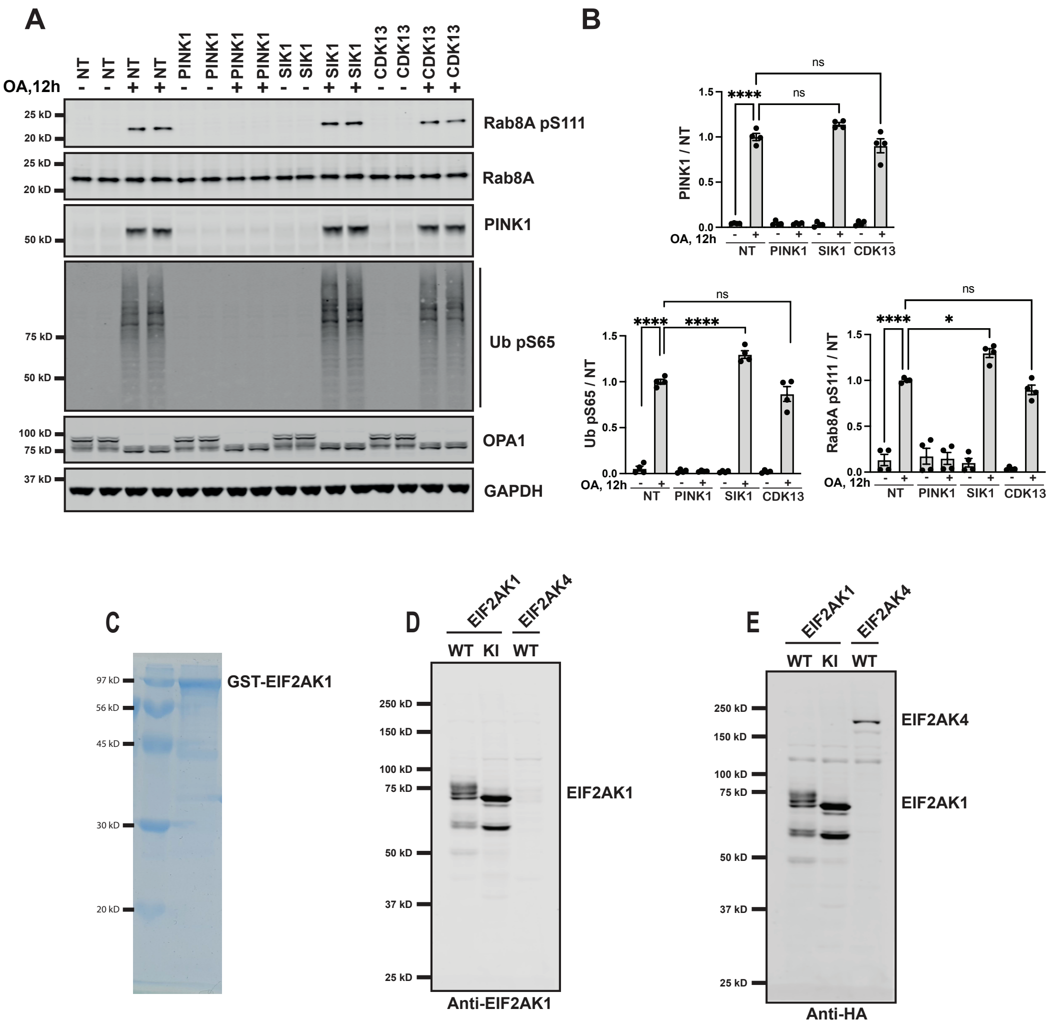

Immunoblot analysis of SIK1 and CDK13 knockdown on PINK1 stabilisation and activation, and generation and validation of polyclonal anti-EIF2AK1 antibody. (**A**) Effect of SIK1 and CDK13 knockdown on PINK1 stabilization and activation. HeLa cells were transfected with independent pooled siRNAs for selected hits (SIK1, CDK13) from the screen and treated with OA in the last 12 h. Cells were lysed and immunoblotted for Ub pS65, PINK1, Rab8A, Rab8A pS111, OPA1, GAPDH and immunoblots were developed using the LI-COR Odyssey CLx Western Blot imaging system. (**B**) Quantification for Ub pS65/GAPDH, PINK1/GAPDH, Rab8A pS111/Rab8A ratio for Fig. A, normalised to the ratio in NT (+OA) samples. (**C**) SDS-PAGE profile of N-terminal GST-tagged EIF2AK1 protein expressed and purified from *E. coli*. (**D, E**) HEK-293 cells transfected with C-terminal HA-tagged EIF2AK1 wild-type (WT) or Kinase-inactive (KI) and wild-type (WT) EIF2AK4 for 24 h. Cells were lysed and immunoblotted using total sheep anti-EIF2AK1 polyclonal antibody generated against full-length human N-terminal GST-tagged EIF2AK1, multiplexed with rat anti-HA antibody for detection of levels of EIF2AK1 and EIF2AK4. Data information: (**B**) All data are mean ± SEM; Statistical significance is displayed as *P ≤ 0.05; **P ≤ 0.01; ***P ≤ 0.001; ****P ≤ 0.0001; ns, not significant. n = 4 technical replicates (2 biological replicates), one-way ANOVA, Tukey’s multiple comparisons test.

Fig. S6.

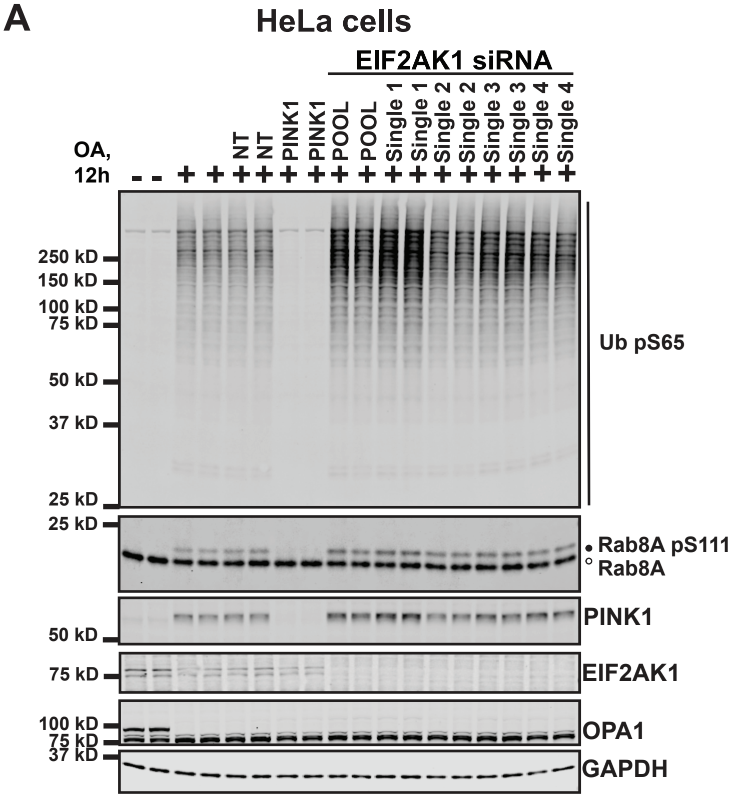

Validation of specificity of siRNA-mediated knockdown of EIF2AK1. HeLa cells were transfected with independent pooled siRNA (Sigma) as well as single siRNAs 1, 2, 3 and 4 (Dharmacon) for EIF2AK1 for 72 h and treated with OA in the last 12 h. Cells were lysed and immunoblotted for Ub pS65, PINK1, Rab8A, EIF2AK1, OPA1, GAPDH and immunoblots were developed using the LI-COR Odyssey CLx Western blot imaging system.

Fig. S7.

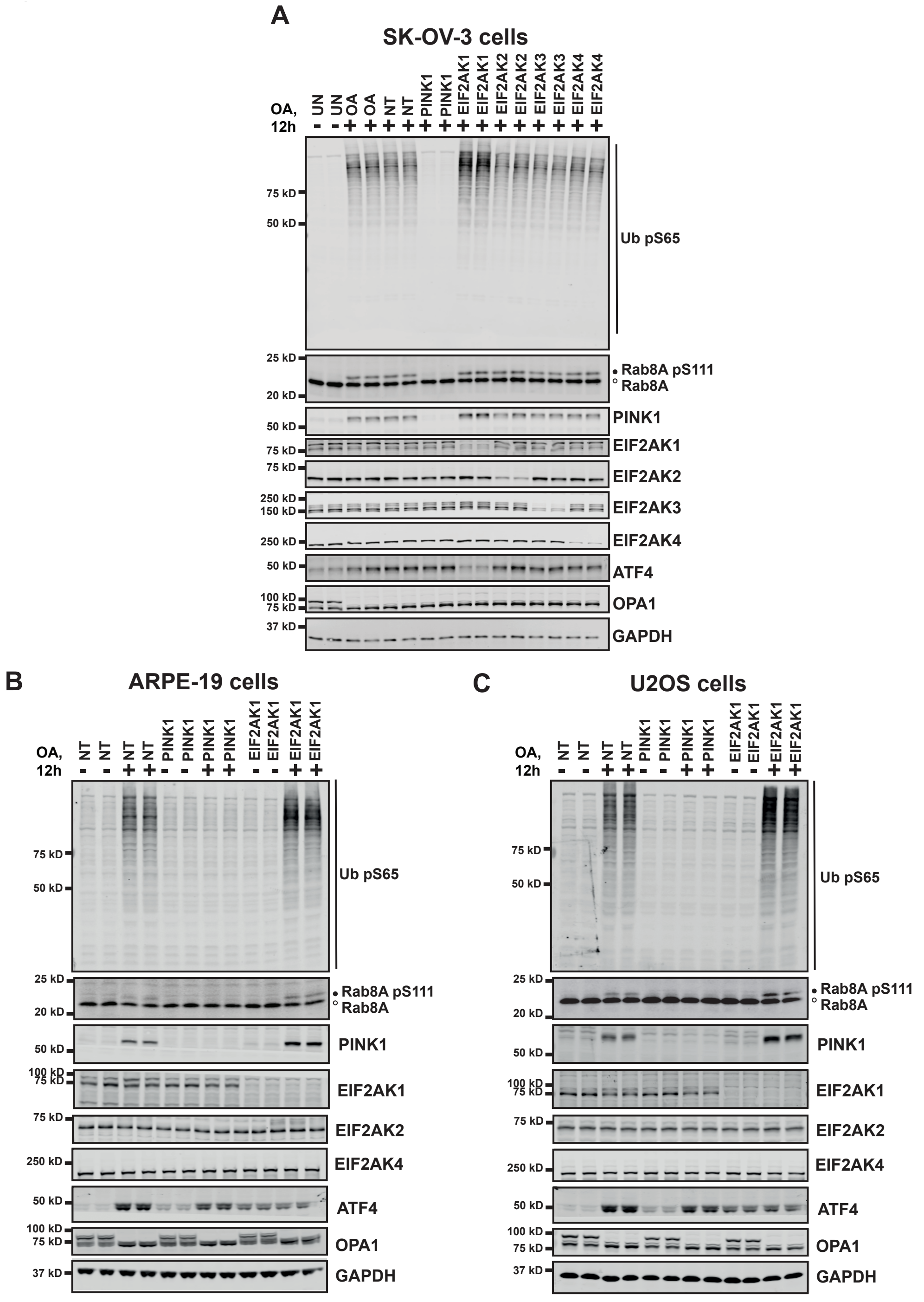

Analysis of EIF2AK1 knockdown on PINK1 stabilisation and activation across a panel of human cell lines. (**A**) SK-OV-3 (**B**) ARPE-19 and (**C**) U2OS cells, either untreated (UN) or, treated with non-targeting control siRNA pool (NT), siRNA pool targeting PINK1, EIF2AK1, EIF2AK2, EIF2AK3, EIF2AK4, for 72 h were treated with OA in the last 12 h. Cells were lysed and immunoblotted for Ub pS65, RAB8A, PINK1, EIF2AK1, EIF2AK2, EIF2AK3, EIF2AK4, OPA1, GAPDH and ATF4 and the immunoblots were developed using the LI-COR Odyssey CLx Western blot imaging system.

Fig. S8.

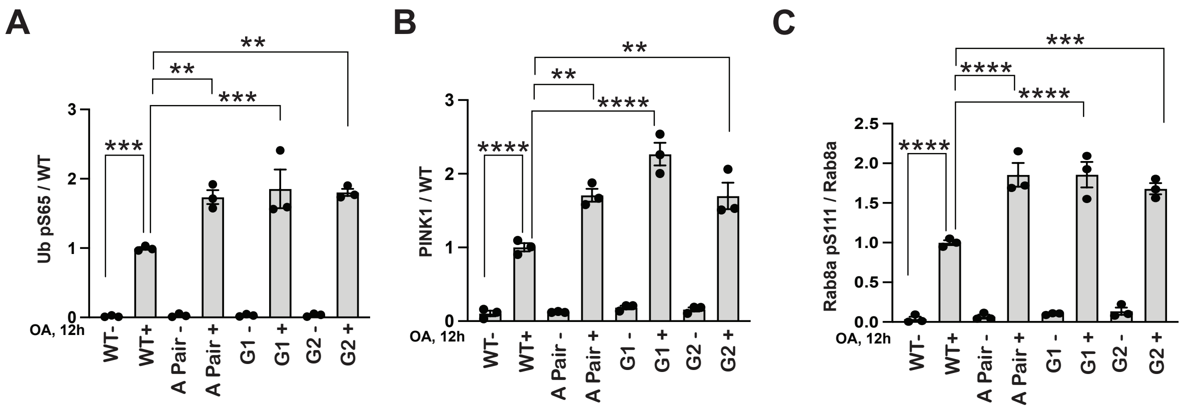

Quantitation of PINK1 stabilisation and activation in EIF2AK1 knockout CRISPR pool lysates. (**A**, **B** and **C**) Quantification for Ub pS65/GAPDH, PINK1/GAPDH and Rab8A pS111/total Rab8A ratio normalised to the ratio in WT+OA samples for Fig. 2F using the Licor Image Studio software.

Data information: (**A**, **B** and **C**) All data are mean ± SEM; Statistical significance is displayed as $*P \leq 0.05$; $**P \leq 0.01$; $***P \leq 0.001$; $****P \leq 0.0001$; ns, not significant. $n = 3$ technical replicates (2 biological replicates), one-way ANOVA, Tukey's multiple comparisons test.

Fig. S9.

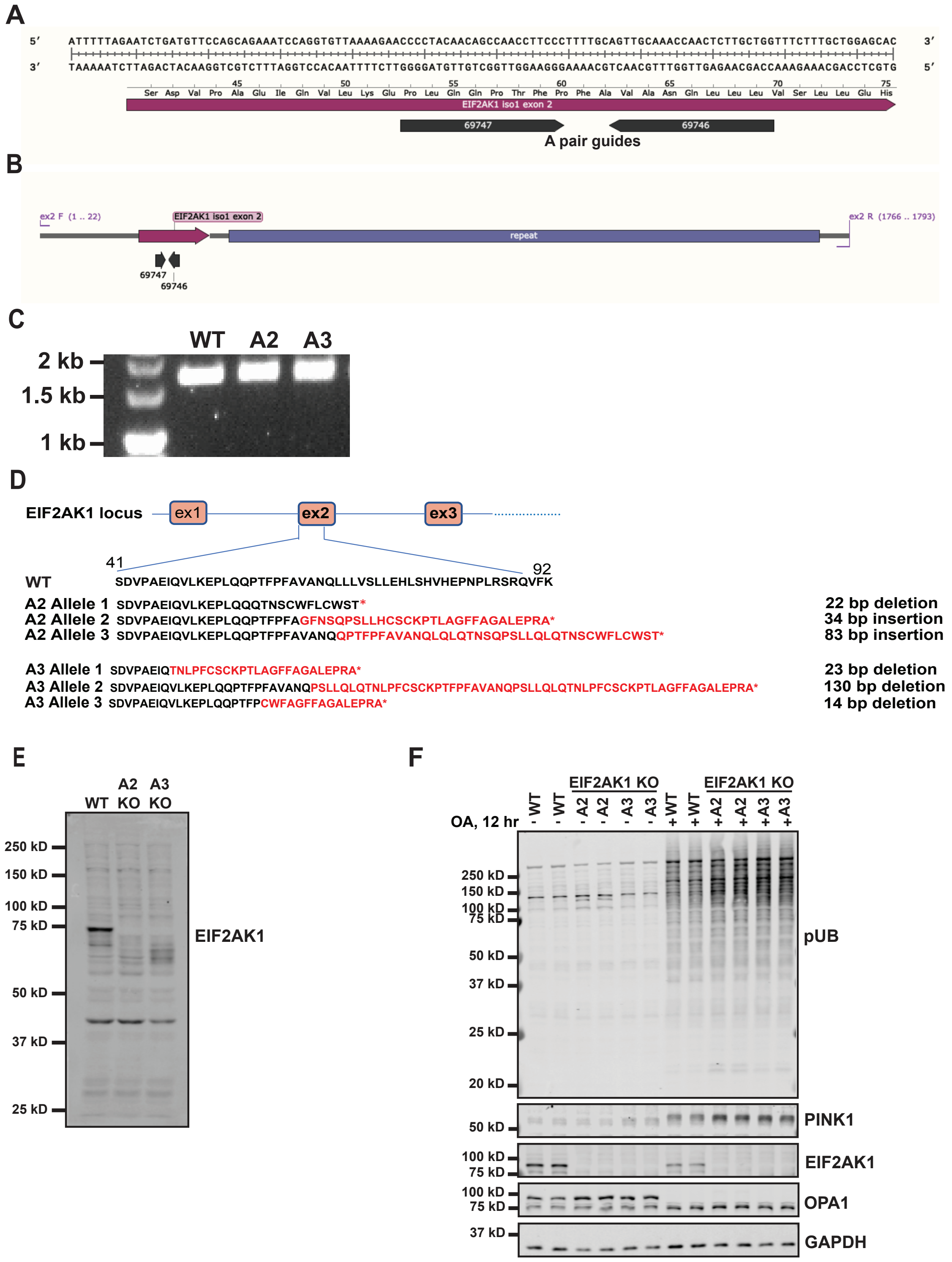

A
5′ ATTTTTAGAATCTGATGTTCCAGCAGAAATCCAGGTGTTAAAAGAACCCCTACAACAGCCAACCTTCCCTTTTGCAGTTGCAAACCAACTCTTGCTGGTTTCTTTGCTGGAGCAC 3′
3′ TAAAAATCTTAGACTACAAGGTCGTCTTTAGGTCCACAATTTTCTTGGGGATGTTGTCGGTTGGAAGGGAAAACGTCAACGTTTGGTTGAGAACGACCAAAGAAACGACCTCGTG 5′
Ser Asp Val Pro Ala Glu Ile Gln Val Leu Lys Glu Pro Leu Gln Gln Pro Thr Phe Pro Phe Ala Val Ala Asn Gln Leu Leu Leu Val Ser Leu Leu Glu His
EIF2AK1 iso1 exon 2
69747
69746
A pair guides
B
ex2 F (1 .. 22)
EIF2AK1 iso1 exon 2
repeat
ex2 R (1766 .. 1793)
69747
69746
C
WT
A2
A3
2 kb
1.5 kb
1 kb
D
EIF2AK1 locus
ex1
ex2
ex3
41
92
WT SDVPAEIQVLKEPLQQPTFPFAVANQLLLVSLLEHLSHVHEPNPLRSRQVFK
A2 Allele 1 SDVPAEIQVLKEPLQQQTNSCWFLCWST*
A2 Allele 2 SDVPAEIQVLKEPLQQPTFPFAGFNSQPSLLHCSCKPTLAGFFAGALEPRA*
A2 Allele 3 SDVPAEIQVLKEPLQQPTFPFAVANQQPTFPFAVANQLQLQTNSQPSLLQLQTNSCWFLCWST*
22 bp deletion
34 bp insertion
83 bp insertion
A3 Allele 1 SDVPAEIQTNLPFCSCKPTLAGFFAGALEPRA*
A3 Allele 2 SDVPAEIQVLKEPLQQPTFPFAVANQPSLLQLQTNLPFCSCKPTFPFAVANQPSLLQLQTNLPFCSCKPTLAGFFAGALEPRA*
A3 Allele 3 SDVPAEIQVLKEPLQQPTFPCWFAGFFAGALEPRA*
23 bp deletion
130 bp deletion
14 bp deletion
E
WT
A2 KO
A3 KO
250 kD
150 kD
100 kD
75 kD
50 kD
37 kD
25 kD
EIF2AK1
F
EIF2AK1 KO
EIF2AK1 KO
OA, 12 hr
- WT
- WT
- A2
- A2
- A3
- A3
+ WT
+ WT
+ A2
+ A2
+ A3
+ A3
250 kD
150 kD
100 kD
75 kD
50 kD
37 kD
25 kD
20 kD
pUB
50 kD
PINK1
100 kD
75 kD
EIF2AK1
100 kD
75 kD
OPA1
37 kD
GAPDH

Generation, characterization, and validation of PINK1 stabilisation and activation in two independent EIF2AK1 knockout clones. (**A**) The sequence of EIF2AK1 in wild-type (WT) HeLa cells at exon 2 is shown and the CRISPR A pair guides used to generate EIF2AK1 Knockout (KO) is highlighted as black arrows. (**B**) The flanking primer position used to confirm successful KO generation is highlighted (**C**) PCR analysis of the wild-type, EIF2AK1 knockout clone A2 and A3 using exon2 flanked forward and reverse primer. PCR products were further cloned and sequenced to confirm homozygous knockout alleles. (**D**) Reference exon 2 translated DNA sequence of wild-type (WT) and sequencing confirming alterations in the HeLa EIF2AK1 KO clone A2 and A3 cell lines has been highlighted. These nucleotide deletion and insertion changes in exon 2 predict a premature stop codon in all three alleles detected in EIF2AK1 KO cells. (**E**) HeLa (wild-type, EIF2AK1 KO Clone A2 and A3 cell extracts were resolved by SDS–PAGE and subjected to Western blotting with EIF2AK1 polyclonal antibody. (**F**) Wild-type (WT) HeLa cells and two independent EIF2AK1 knockout HeLa cells clones A2, A3 that had all been through in parallel,single-cell sorting, and expansion were treated with OA for 12 h. Cells were lysed and immunoblotted for Ub pS65, PINK1, EIF2AK1, OPA1, GAPDH and the immunoblots were developed using the LI-COR Odyssey CLx Western blot imaging system.

Fig. S10.

A

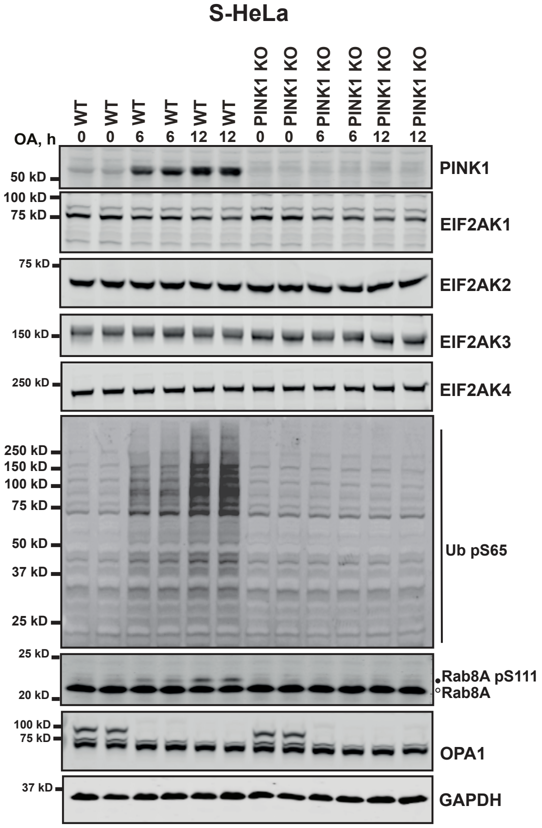

Analysis of EIF2AK1 expression in S-HeLa PINK1 knockout cells. Wild type (WT) and PINK1 knockout S-HeLa cells were treated with OA for 6 and 12 h. Immunoblot of EIF2AK1, EIF2AK2, EIF2AK3, EIF2AK4, Ub pS65, PINK1, Rab8A, OPA1 and GAPDH were developed using the LI-COR Odyssey CLx Western blot imaging system.

Fig. S11

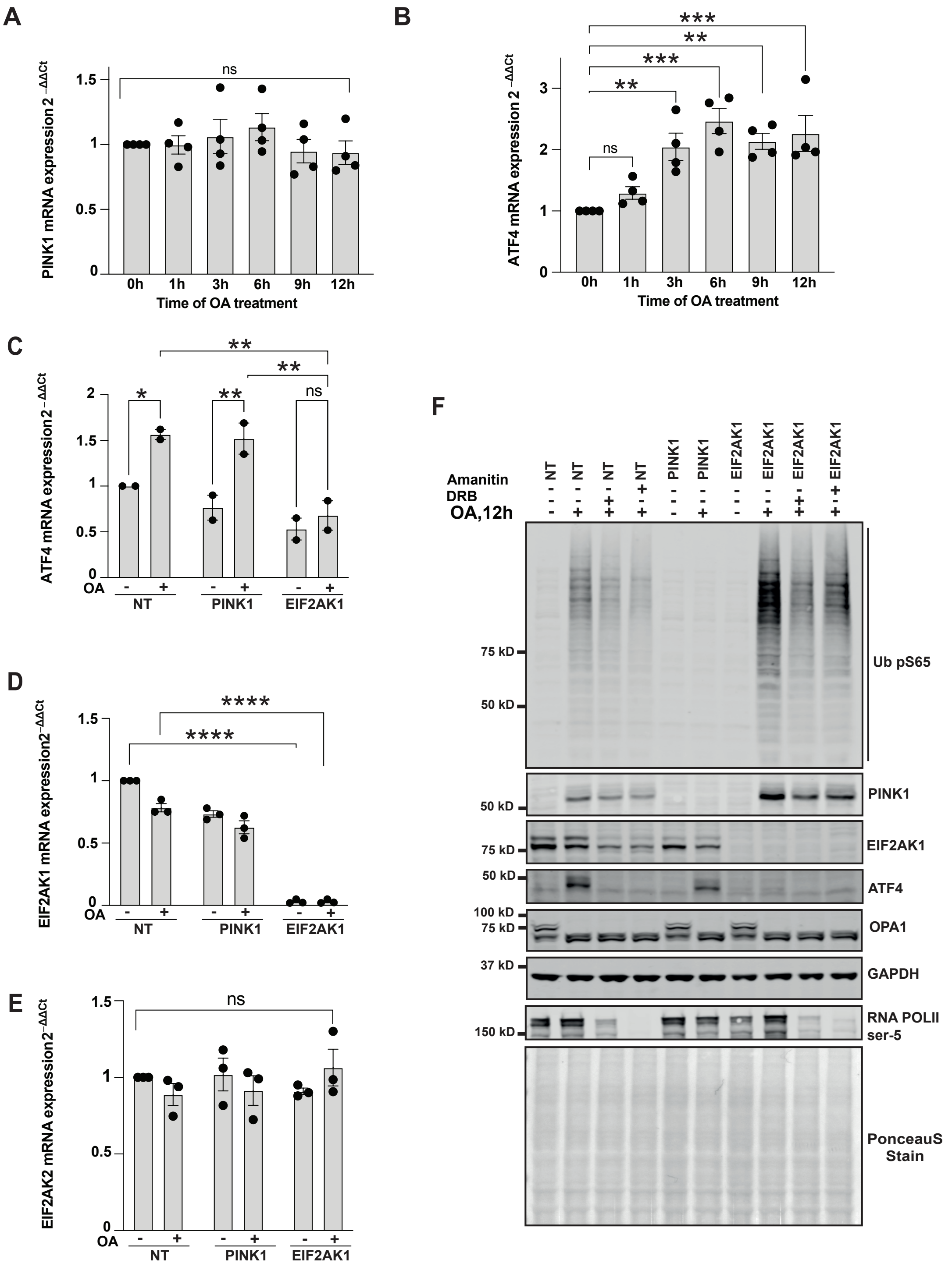
A
PINK1 mRNA expression 2 −ΔΔCt
ns
0h 1h 3h 6h 9h 12h
Time of OA treatment
B
ATF4 mRNA expression 2 −ΔΔCt
***
**
***
**
ns
0h 1h 3h 6h 9h 12h
Time of OA treatment
C
ATF4 mRNA expression 2 −ΔΔCt
**
**
*
**
ns
OA
NT
PINK1
EIF2AK1
D
EIF2AK1 mRNA expression 2−ΔΔCt
****
****
OA
NT
PINK1
EIF2AK1
E
EIF2AK2 mRNA expression 2−ΔΔCt
ns
OA
NT
PINK1
EIF2AK1
F
Amanitin
DRB
OA,12h
NT NT NT NT PINK1 PINK1 EIF2AK1 EIF2AK1 EIF2AK1 EIF2AK1
75 kD
50 kD
Ub pS65
50 kD
PINK1
75 kD
EIF2AK1
50 kD
ATF4
100 kD
75 kD
OPA1
37 kD
GAPDH
150 kD
RNA POLII ser-5
PonceauS Stain

Transcriptional regulation of PINK1 and ATF4 in EIF2AK1 silenced cells. (**A**) Relative mRNA levels of PINK1 measured by RT-PCR in HeLa cells treated with OA for 1, 3, 6, 9 and 12 h. (**B**) as in A, Relative mRNA levels of ATF4 measured by RT-PCR in HeLa cells treated with OA for 1, 3, 6, 9 and 12 h. (**C**) Relative mRNA levels of ATF4 measured by RT-PCR in Hela cells treated with non-targeting control siRNA pool (NT), siRNA pool targeting PINK1 or, EIF2AK1for 72 h and with OA in the last 12 h. (**D**) Relative mRNA levels of EIF2AK1 measured by RT-PCR in Hela cells treated with non-targeting control siRNA pool (NT), siRNA pool targeting PINK1 or, EIF2AK1for 72 h and with OA in the last 12 h. (**E**) Relative mRNA levels of EIF2AK2 measured by RT-PCR in HeLa cells treated with non-targeting control siRNA pool (NT), siRNA pool targeting PINK1 or, EIF2AK1for 72 h and with OA in the last 12 h. (**F**) Immunoblot analysis for HeLa cells treated with non-targeting control siRNA pool (NT), siRNA pool targeting PINK1 or, EIF2AK1for 72 h. Co-treated with OA and translation inhibitors DRB and alpha-Amanitib or, OA alone in the last 12 h. Immunoblot of Ub pS65, PINK1, EIF2AK1, OPA1, GAPDH, ATF4, RNA POLII phospho-S5 (DRB treatment control) and total protein as visualized by PonceauS staining. Data information: (**A-E**) All data are mean ± SEM; Statistical significance is displayed as $*P \leq 0.05$; $**P \leq 0.01$; $***P \leq 0.001$; $****P \leq 0.0001$; ns, not significant. n = 3 biological replicates. (**A**) one-way ANOVA, Tukey's multiple comparisons test, (**B**) one-way ANOVA, Sidak's multiple comparisons test and (**C-E**) two-way ANOVA, Uncorrected Fisher's LSD multiple comparisons test.

Fig. S12

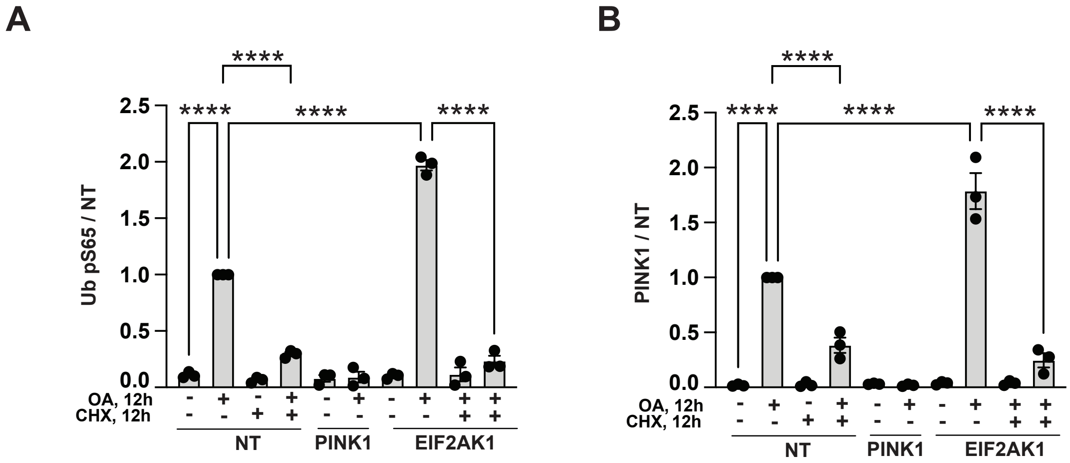

Quantitation of translation inhibition of PINK1 upon CHX treatment (**A**, **B**) Quantification for Ub pS65/GAPDH and PINK1/GAPDH ratio normalised to the ratio in OA alone samples for Fig. 4E using the Licor Image Studio software. Data information: (A and B) All data are mean ± SEM; Statistical significance is displayed as *P ≤ 0.05; **P ≤ 0.01; ***P ≤ 0.001; ****P ≤ 0.0001; ns, not significant. n = 3 biological replicates, one-way ANOVA, Tukey's multiple comparisons test.

Fig. S13.

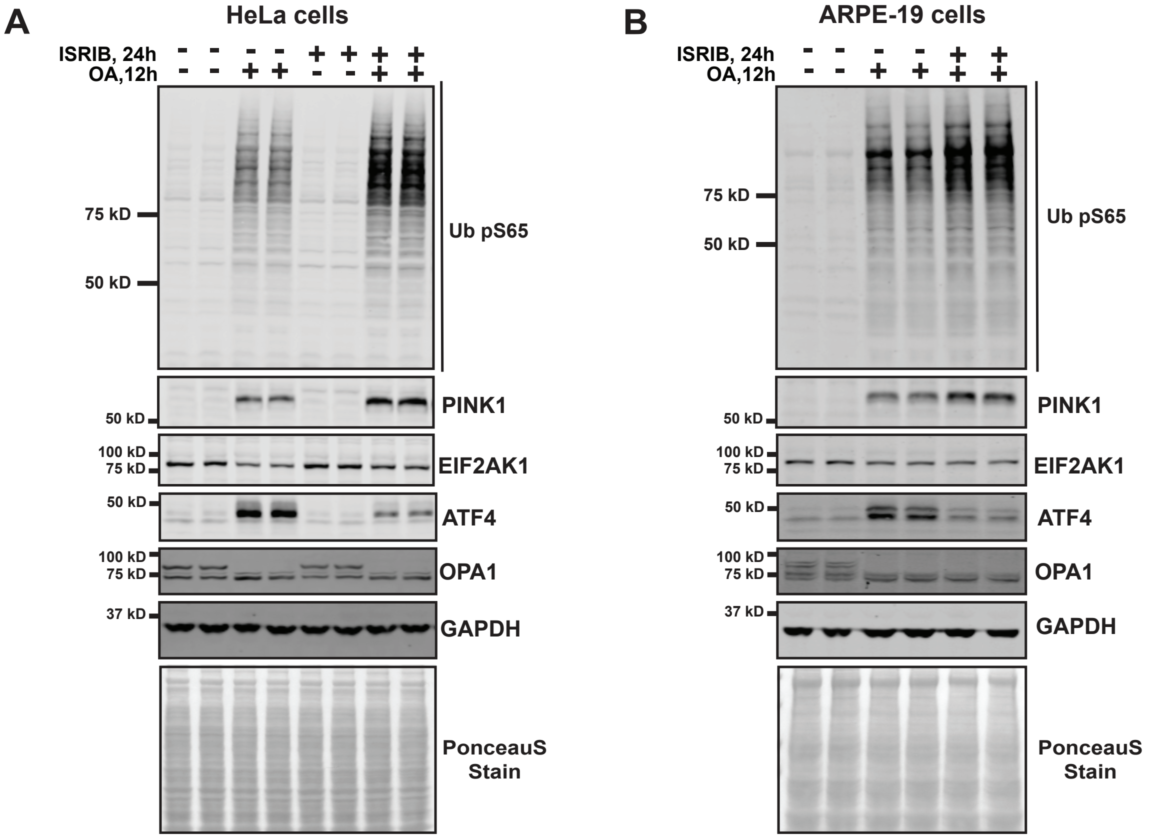

Validation of chemical inhibition of the ISR by ISRIB in HeLa and ARPE-19 cells. (**A**) HeLa cells seeded in a 10 cm dish, were treated with 300nmol of ISRIB for 24 h with and without co-treatment of OA for 12 h or, OA alone for 12 h. Representative Immunoblot of Ub pS65, PINK1, EIF2AK1, ATF4, OPA1, GAPDH and total protein as visualized by PonceauS staining. (**B**) As in A, ARPE-19 cells were co-treated with 300nmol of ISRIB for 24 h with OA for 12 h or, OA alone for 12 h. Representative Immunoblot of Ub pS65, PINK1, EIF2AK1, ATF4, OPA1, GAPDH and total protein as visualized by PonceauS staining.

Fig. S14.

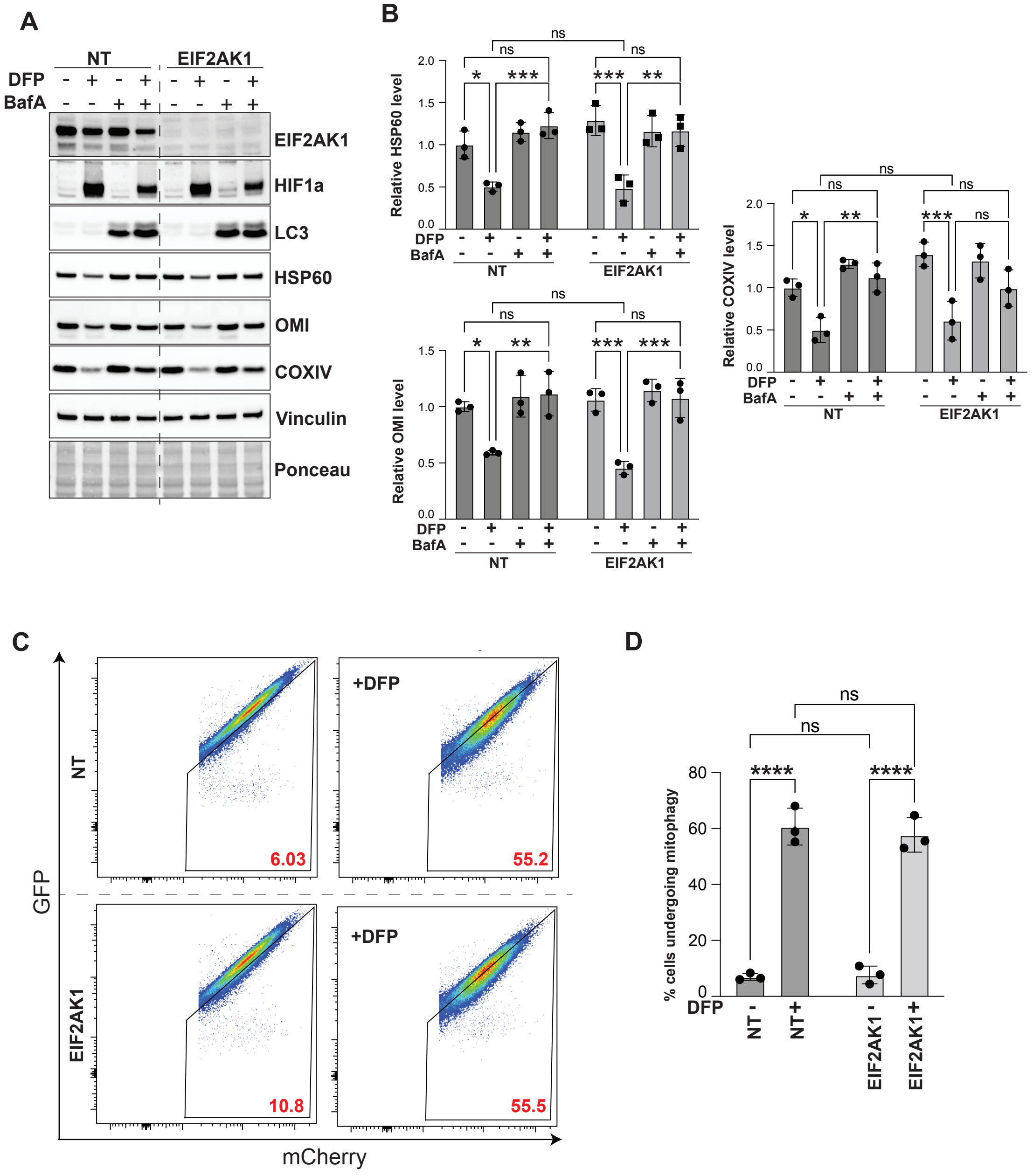

Investigation of role of EIF2AK1 in DFP-induced mitophagy in cells (**A**, **B**) Representative immunoblots (**A**) and quantifications (**B**) of the indicated proteins in lysates of ARPE-19 cells stably expressing the *mito*-QC reporter and HA-Parkin, transfected with non-targeting siRNA (NT) or siRNA targeting EIF2AK1. 2 days post transfection, cells were treated with 1 mM DFP and/or with 50 nM Bafilomycin A (BafA) for an additional 24 h. (**C**, **D**) Flow cytometry analysis of mitophagy in cells treated as in A.(**C**)Representative dot plots are shown after analysing GFP and mCherry signals. The percentage of cell underdoing mitophagy is indicated in bold red on each dot plot. Data information: Overall data are mean ± s.d.; Statistical significance is displayed as *$P \leq 0.05$; **$P \leq 0.01$; ***$P \leq 0.001$; ****$P \leq 0.0001$; ns, not significant. (**B**) n = 3 biological replicates, two-way ANOVA, Tukey's multiple comparisons test. (**D**) n = 3 biological replicates, two-way ANOVA, Sidak's multiple comparisons test.

Table S1

Licor immunoblot quantification of siRNA kinome screen

Table S2

Dharmacon siRNA kinase library used in screen

Table S3

List of antibodies
